# Quantifying intracellular mechanosensitive response upon spatially defined mechano-chemical triggering

**DOI:** 10.7554/eLife.107220

**Published:** 2026-06-17

**Authors:** Elaheh Zare-Eelanjegh, Renard TM Lewis, Ines Lüchtefeld, Ulrike Kutay, Tomaso Zambelli

**Affiliations:** 1 Laboratory of Biosensors and Bioelectronics, Institute for Biomedical Engineering, ETH Zurich Zurich Switzerland; 2 Department of Biology, Institute of Biochemistry, ETH Zurich Zurich Switzerland; Yale School of Medicine United States; Universitat Pompeu Fabra Spain

**Keywords:** mechanotransmission, nuclear mechanoresponse, endoplasmic reticulum mechanoresponse, lamins, cytoskeleton, HeLa cells, Other

## Abstract

The mechanotransduction process relies on the interaction of mechanical and biochemical cues, transmitting cellular forces to intracellular organelles to activate biochemical pathways and elicit responses. This involves mechanoresponsive components like actin filaments, microtubules (MTs), and the lamin meshwork. Fluidic force microscopy (FluidFM), a force-controlled micropipette, allows for the manipulation of intact cells mechanically and chemically, providing a novel approach to study mechanotransmission in cells in situ. FluidFM combined with fluorescence lifetime imaging microscopy (FLIM) enables high-resolution mapping of intracellular tension dynamics. Here, we used cells with varying nuclear lamina compositions to explore the lamina’s role in initiating mechanoresponse to external cues. We found that A- and B-type lamins trigger nuclear mechanoresponse distinctly, with A-type lamins contributing to nuclear elasticity, whereas B-type lamins influence viscous response. Moreover, MTs underwent mechanical adaptation and assisted in releasing the tension in lamin A/C knockout (KO) cells, contrasting with healthy cells where MTs aid in preserving the tension locally rather than transferring it. This research provides insights into the dynamic mechanoresponse of cellular components and supports targeted therapies for mechanical stress-related diseases.

## Introduction

Mechanical forces regulate cell behavior and function. The cytoskeleton, particularly the actinomyosin network, acts as a primary receptor and transmitter of these signals (Faria, 2018; Jonas and Duschl, 2010). Biological tissues and organs are influenced by these mechanical signals at the cellular level (Li et al., 2022b).

Moreover, cells coordinately transmit external forces into the intracellular organelles, activate biochemical pathways, and eventually elicit corresponding cellular responses (Swaminathan and Gloerich, 2021; Mathieu and Manneville, 2019). This relies on interconnected mechanosensory systems, including ion channels, membrane receptors, cytoskeletal components, and the Linker of Nucleoskeleton and Cytoskeleton (LINC) complex that bridges the cytoskeleton to the nucleus (Lityagina and Dobreva, 2021). Additionally, the nucleus, a major mechanosensitive organelle, can directly sense large-scale forces resulting in nuclear deformations and gene expression changes via a process called ‘nuclear mechanotransduction’ (Kirby and Lammerding, 2018). Other organelles such as the endoplasmic reticulum (ER) (Kim et al., 2015), mitochondria (Mahecic et al., 2021), and Golgi apparatus (Guet et al., 2014) can also sense transmitted forces. The nuclear lamins, intermediate filaments lining the inner side of the inner nuclear membrane, likely collaborate with the LINC to regulate nuclear tension and mechanotransduction (Donnaloja et al., 2020; Zheng et al., 2022). Forces convert into biochemical cues at the nuclear envelope (NE) through phosphorylation of lamin A and emerin, a mechanosensitive inner nuclear membrane protein that associates with lamin A/C (Wintner et al., 2020). The general belief is that viscoelastic responses of the nucleus to physiological forces are governed by the interactions between A- and B-type lamins as well as chromatin (Patil and Sengupta, 2021). Understanding the mechanical roles of lamin A/C and lamins B1/B2 will enhance our knowledge of nuclear deformation in health and disease. Notably, different mechanotransduction pathways crosstalk and interplay with biochemical signals (e.g., growth factors) to elucidate specific cell behaviors (Dalton and Lemmon, 2021). Disruption of biomechanical homeostasis and failure of cells to appropriately transmit the mechanical stimuli into the requisite biochemical pathways can lead to diseases such as arthritis, atherosclerosis, and cancer (Hang et al., 2021; Broussard et al., 2017).

Several methods have been developed to explore single-cell mechanics and mechanotransmission including atomic force microscope (AFM), optical tweezer, magnetic tweezer, biomembrane force probes, traction force microscope, optogenetic mechanostimulators, and DNA-based mechanical sensors (Blakely et al., 2014; Tian et al., 2022). Recently, a time-shared optical tweezer microrheology method (TimSOM) that measures viscoelastic properties of cytoplasm, NE, and nucleoplasm in living systems has been reported (Català-Castro et al., 2025). However, these methods often face limitations in measuring intracellular forces at the molecular level within the cell’s natural state (Li and Jan, 2025). For instance, the recently developed TimSOM requires embedding tracer beads inside cells or embryos and yields averaged measurements over small regions rather than pinpointing single molecular-scale forces. To overcome such constraints, FRET (Freikamp et al., 2016; Eder et al., 2017) and FLIM-based (Goujon et al., 2019; Colom et al., 2018) tension sensors have been engineered for studying intracellular mechanosensing at the molecular level. FRET detects energy transfer between two fluorophores in proximity, indicating molecular interactions and conformational changes within biological systems. Unlike intensity-based FRET methods, FLIM measures the lifetime of fluorescent molecules excited by a laser pulse, offering a quantitative signal for molecular interactions independent of reporter molecule concentration. To delve into the interplay of biochemical and mechanical aspects in cellular behaviors, we require tools that can sensitively stimulate living cells both mechanically and chemically in their natural state, while observing intracellular dynamics. Furthermore, these methods should quantify minute forces with superior spatial and temporal resolution.

Fluidic force microscopy (FluidFM), a micropipette configured on an AFM, addresses this challenge (Meister et al., 2009). The FluidFM micro channeled-cantilever with a nano-aperture, connected to a fluidic pressure controller, enables quantitative injection of impermeable molecules into subcellular compartments of living cells (Guillaume-Gentil et al., 2016; Guillaume-Gentil et al., 2013; Guillaume-Gentil et al., 2017; Li et al., 2022a). Such injections can be realized as biochemical triggering of the cellular events with spatial and temporal resolution while preserving cell viability. Combining FluidFM with optical readouts like FLIM-based molecular sensors enables simultaneous manipulation of intact cells and quantifying their responses.

In this work, we first used FluidFM combined with the FLIM for in situ mechanical (i.e., indentation) and chemical (i.e., drug injection) triggering of cells with simultaneous intracellular tension mapping at the NE and ER. Further, mechanoresponsive crosstalk between different organelles within the cell including the nucleus, ER, and cytoskeleton. For chemical interference, we injected cytochalasin D (CytoD) and/or nocodazole (Noco) to inhibit the actin and/or microtubule (MT) network, respectively. Next, we exploited cell lines in which nuclear lamins were perturbed, that is, wild-type HeLa (WT), lamin A/C gene (*LMNA*) KO, *LMNB1* and *LMNB2* (LMNB)-knockdown (KD), and *LMNA* KO+LMNB-KD and subjected to mechano-chemical stimulation and quantification of tension changes.

## Results and discussion

We had previously reported the development of a FluidFM-FLIM system based on a customized stage for mounting our AFM head on top of a confocal inverted microscope to investigate the effect of the plasma membrane tension on the opening of mechanosensitive ion channels (Lüchtefeld et al., 2024). Here, we used HeLa cells stained with ER Flipper-TR, a tension reporter dye which selectively labels the ER and nuclear membrane constituting a continuous membrane network (Goujon et al., 2019). ER Flipper-TR measures the membrane tension changes through alterations in its fluorescence lifetime and serves as a quantitative readout for the tension responses to the external stimuli within living cells. FLIM signals were recorded before, during, and after the mechanical or mechano-chemical stimulus facilitated by FluidFM.

### Mechanical stimulation of the nucleus: impact of probe apex shape on cellular mechanoresponse

**Figure 1.**
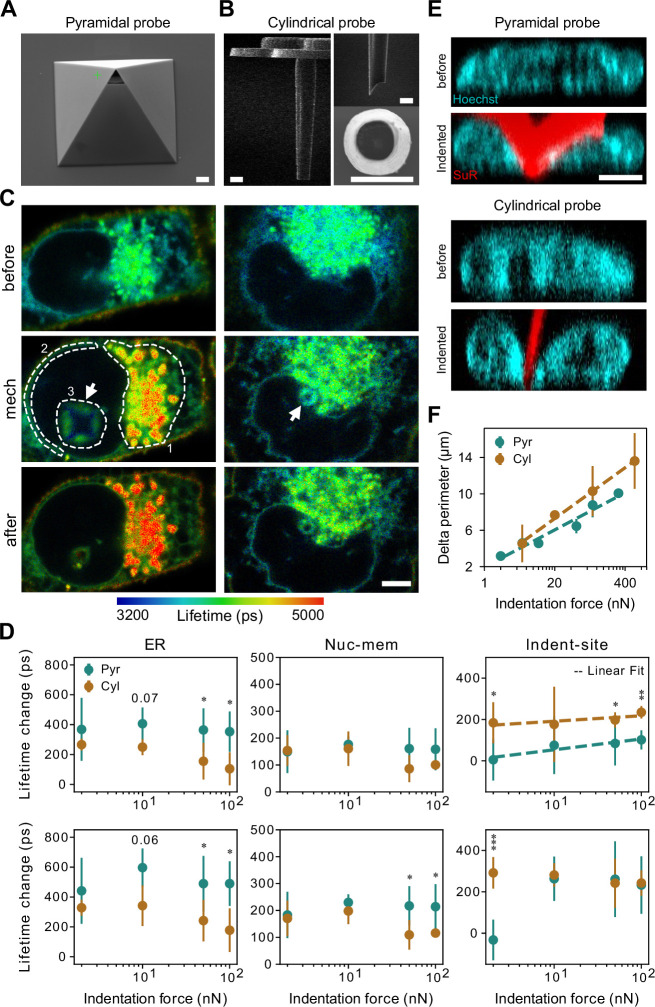
FluidFM stimulation combined with FLIM readout for measuring subcellular mechanoresponse upon mechanical stimulation of nuclei. SEM micrographs of a pyramidal probe (**A**) and a cylindrical probe (**B**) before (left) and after FIB-cut (top right). The top view of the cylinder is also shown in panel B. Scale bars represent 1 µm. (**C**) Lifetime images of HeLa cells stained with molecular tension probe (ER Flipper-TR) before (top), during (middle), and after (bottom) indentation at 100 nN, with pyramidal (left panel) and sharpened cylindrical probes (right panels). Arrows denote the indentation site when either pyramidal or cylindrical probes are used. Dashed lines indicate a representative selection of regions of interest (ROIs), encompassing (1) the ER, (2) nuclear membrane (Nuc-mem), and (3) the area around the indentation site (Indent-site). Scale bar represents 5 µm. (**D**) Change in lifetime during (top panel, ‘mech’ in C) and after (bottom panel) mechanical stimulus with varied indentation forces at different ROIs: ER, nucleus membrane (Nuc-mem), and incident indentation site (‘Indent-site’) (mean ± SD, *N* ≥ 2, *n* ≥ 4, ***p < 0.001, **p < 0.01, *p < 0.05, reported p-values of 0.05 ≤ p < 0.1, *t*-test). For mechanical stimulation, pyramidal (‘Pyr’) and cylindrical (‘Cyl’) probes with similar stiffness (2 ± 0.5 Nm^−1^) were utilized. (**E**) Lateral view of nucleus before (top panels) and during indentation (bottom panels) (indicated by ‘mech’ in panel C) at 100 nN using pyramidal and cylindrical probes. Nuclei are stained with Hoechst dye (blue) and probes are filled with sulforhodamine dye (SuR, red). Scale bar represents 5 µm. (**F**) Change in perimeter of nucleus measured from confocal images at varied indentation forces, with pyramidal and sharpened cylindrical probes (mean ± SD, *N* = 2, *n* ≥ 3).

The geometry of an AFM probe determines how a force is spatially applied on a cell surface (Yang et al., 2021). Different probe shapes cause varying membrane deformations, activating diverse mechanoresponses through mechanical involvement of various organelles. The cellular cytoskeleton plays crucial roles in distributing and absorbing mechanical forces to maintain cellular integrity and function. Cylindrical and pyramidal FluidFM probes were used for indenting and puncturing the nucleus under varied forces (Figure 1A, B). Cylindrical FluidFM probes were sharpened using a focused ion beam (FIB) to facilitate membrane rupture with minimal stress (Figure 1B, see Methods section). In parallel, lifetime images of ER Flipper-TR stained HeLa cells were obtained using a FLIM detector integrated into a confocal laser scanning microscope (Figure 1C). The images, color-coded for clarity, report the conformational shifts in the ER Flipper-TR molecule. Higher lifetime values (red color) indicate increased membrane tension.

**Figure 1—figure supplement 1.**
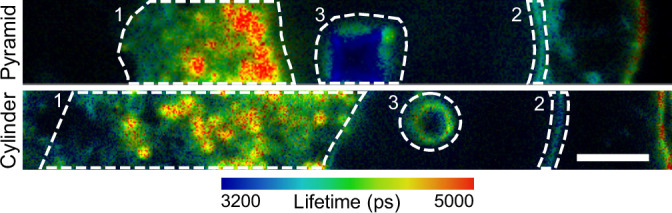
Selection of regions of interest (ROIs) of (1) the ER, (2) nuclear membrane (Nuc-mem), and (3) a spot around the indentation site (Indent-site) using pyramidal (top) and cylindrical (bottom) probes. Representative lifetime images of a HeLa cell stained with ER Flipper-TR dye are shown. Scale bar represents 5 µm.

**Figure 1—figure supplement 2.**
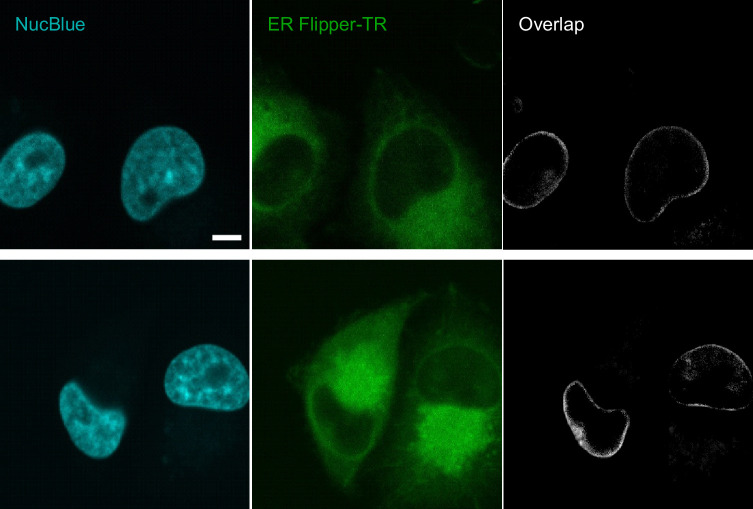
Co-staining HeLa cells with NucBlue (targeting DNA inside the nucleus) and ER Flipper-TR dye. The significant overlap signals obtained provide confirmation of the precision of the designated region of interest (ROI) as representing the nuclear membrane in this investigation. Scale bar represent 5 µm.

Subsequently, different ‘regions of interest’ (ROIs) were manually selected for the ER, nuclear membrane (Nuc-mem), and the area around the indentation site (Indent-site) (Figure 1—figure supplements 1 and Figure 1C, middle), and their lifetime values were analyzed. Mean lifetime values within each ROI were normalized by subtracting the initial values for each cell (see Methods section and Figure 1D). The average lifetime values at the ER showed greater tension increase with pyramidal probes compared to cylindrical probes across a range of indentation forces (10–100 nN) (Figure 1D, top left). The ER retains this higher tension even after retracting the probe and removal of the external mechanical stimulus (Figure 1D, bottom left). At the NE, both pyramids and cylinders impose the same amount of stress during mechanical stimulus (Figure 1D, top middle). Yet, following the removal of the probe terminating the mechanical perturbation, pyramidal probes reveal increased tension at NE at higher indentation forces of 50 and 100 nN in contrast to the cylinders (Figure 1D, top middle). At the NE, a decrease in lifetime or relaxation in tension occurred between 10 and 50 nN indentation, observed only with cylindrical probes. Since this is within the expected range of indentation force during membrane rupture, the observed relaxation in tension is related to membrane rupture and cylinder insertion in HeLa cells. Although pyramidal probes are also expected to puncture the cell membrane and subsequently the NE upon indenting cells targeting their nucleus with increasing forces (i.e., around 100 nN for HeLa cells; Guillaume-Gentil et al., 2013), their insertion phenomena within the membranes is not instantaneous and thus not detectable within the graph demonstrating the quantified tension upon indentation (Figure 1D, top middle). Sharpened cylindrical probes elicit higher tension locally at the indentation site when compared to pyramids during indentation (Figure 1D, top right). This is due to the cylindrical geometry leading to a smaller area where the inserting force is employed, increasing the pressure locally while minimizing the disturbance to other organelles such as the ER. Hence, sharpened cylindrical probes exert a lower overall tension on the cells during injection. Immediately following the retraction of cylindrical probes, the tension remains constant or slightly reduces. But for pyramidal probes, surprisingly, the lifetime increases within all ROIs immediately following the retraction. This suggests an additional mechanical stress upon the withdrawal of pyramidal probes.

Lastly, to assess the origin of higher tension imposed locally by cylindrical probes compared to pyramidal ones, the deformation of cell nuclei was quantified during the indentation process using lateral images acquired by the confocal microscopy (Figure 1E). For this, FluidFM probes were filled with sulforhodamine (SuR) dye (red), and the nuclei, stained with Hoechst (blue), were subjected to indentation at varied forces while acquiring z-stack images before and during stimulus. Then, the lateral views were obtained from z-stack images, and the nucleus perimeter was measured before and during indentation for both pyramidal and cylindrical probes (Figure 1F). As expected, cylinders introduce higher deformation of cell nuclei with maximum deformation around the apex (Figure 1E). This rationalizes the higher lifetime values reported for the indentation site for cylinders compared to pyramids (Figure 1D, top right).

Collectively, the use of different probe shapes for mechanical stimulation of the nucleus revealed a distinct tension distribution across varied subcellular compartments. While tension was higher around the cylindrical probe, a higher tension at the ER was observed when pushing with a pyramidal probe. This could be attributed to the greater total volume displacement caused by the broader apex of pyramidal probes compared to the sharper tips of cylindrical ones. Furthermore, tension was increased in the ER and the NE of cells stimulated by the pyramids upon removal of probes, which was not the case for the cylinders. Since the cytoskeleton is essential for sensing such mechanical stimuli and for the downstream responses (e.g., transcription), we further explored whether chemically induced depolymerization of actin filaments affects the response of cells to mechano-stimulation, combining nucleus indentation and CytoD injection.

### Disrupting actin filament dynamics by mechano-chemical triggers inhibits their active support of the stressed nucleus

**Figure 2.**
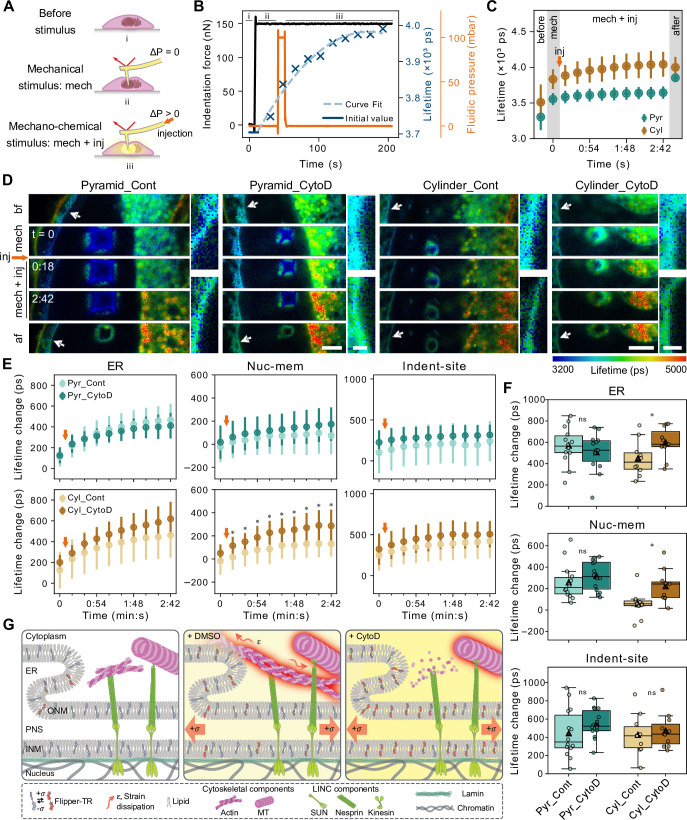
FluidFM-FLIM mechano-chemical stimulation of HeLa cell nuclei using pyramidal and cylindrical probes. (**A**) Schematic showing the mechanotransduction at LINC complexes before (top) and after external stimulus: mechanical (middle) or mechano-chemical (bottom) stimulus. ∆*P* represents the fluidic pressure difference between the probe reservoir and the aperture. When this pressure difference is positive, it indicates that the injection mode of FluidFM is being utilized. (**B**) Concurrent readout of atomic force microscope (AFM)-measured indentation force, ER Flipper-TR lifetime, and fluidic pressure pulse for injection over time. For assured injection, 150 nN was chosen as the set point for indentation using both probe geometries. Parts i, ii, and iii on the graph in B and the schematic in A represent: (i) before stimulus, (ii) during mechanical stimulus when the probe has reached the set point on the cell without fluidic pressure application, and (iii) during mechano-chemical stimulus when the probe remains in contact with the cell at the set point, and a fluidic pressure pulse is applied for drug (or control) injection. (**C**) Absolute lifetime values before, during, and after injecting HeLa cells with CytoD with pyramidal (‘Pyr’) and cylindrical (‘Cyl’) probes at indentation site (mean ± SD, *N* ≥ 3, *n* ≥ 11). The orange arrow indicates the injection pulse at the specified time in the mechano-chemical triggers. (**D**) Time-lapse lifetime images of ER Flipper-TR-stained HeLa cells before, upon indentation (‘mech’), after indentation plus injection (‘mech + inj’) of either DMSO (as control) or 50 µM CytoD, and after retracting pyramidal (left) and cylindrical (right) probes. The numbers represent time (min:s), with time 0 corresponding to the indented state (mechanical stimulus, noted as ‘mech’). Inserts represent magnified nuclear membrane at locations specified with arrows. Scale bars in time-lapse images and inserts represent 5 and 1 µm, respectively. (**E**) Change in lifetime at different regions of interest (ROIs): ER, nucleus membrane (Nuc-mem), and indentation site (‘Indent-site’) when mechano-chemically challenged by either CytoD or Control using pyramidal (top row) and cylindrical (bottom row) probes (mean ± SD, *N* ≥ 3, *n* ≥ 11, ***p < 0.001, **p < 0.01, *p < 0.05, reported p-values of 0.05 ≤ p < 0.1, *t*-test). Orange arrows indicate the injection pulse at the specified time in the mechano-chemical process. (**F**) Boxplots showing lifetime change upon retracting probes from the injected cells at different ROIs: ER, Nuc-mem, and indentation site. Mean values are shown with triangles (*N* ≥ 3, *n* ≥ 11, ***p < 0.001, **p < 0.01, *p < 0.05, reported p-values of 0.05 ≤ p < 0.1, ns: not significant, *t*-test). (**G**) Schematic represents the tension report of ER Flipper-TR dye inside the membrane of the nucleus and the ER before (left panel) and after mechano-chemical triggering of the nucleus with DMSO (as control, middle panel) or CytoD (right panel). In contrast to control cells, delivery of CytoD induces more accumulated tension (i.e., FLIM data) at the nuclear membrane and the ER because it disrupts load-bearing actin component of cytoskeleton. ONM: outer nuclear membrane, INM: inner nuclear membrane, PNS: perinuclear space, and σ: tension exerted by indenting the nucleus using FluidFM probes.

The dynamics of cytoskeletal and nuclear actin, whether in monomeric (G-actin) or filamentous (F-actin) forms, affects both the sensing and response to mechanical stimuli, as well as the resulting transcriptional processes (Ulferts et al., 2021; Dupont and Wickström, 2022). The mechanosensitive LINC complex in tandem with cytoskeletal components regulates nuclear function and protects the nucleus from excessive mechanical stress. CytoD disturbs actin filament dynamics mainly by binding to G-actin and inhibiting filament assembly (Gokhin et al., 2015). We investigated whether CytoD interruption of the actin network alters the tension load on the nucleus, thus modifying the nuclear mechanoresponse. Therefore, actin filaments were chemically disrupted by a mechano-chemical stimulation through indenting the nucleus and concurrently injecting CytoD in a spatio-temporally defined fashion (Figure 2A, B). Since CytoD is smaller in size than the passive diffusion limit of nuclear pore complexes, it distributes throughout the nucleus and cytosol, interacting with both nuclear and cytoskeletal actin, thereby influencing overall cell structure and its mechanoresponsive behavior. While CytoD is believed to permeate cell membranes, its active intracellular injection allows for real-time tracking of dynamic responses. Additionally, when quantitatively injected, the amount taken up by different cells is consistently controlled, regardless of variations in cell responses.

**Figure 2—figure supplement 1.**
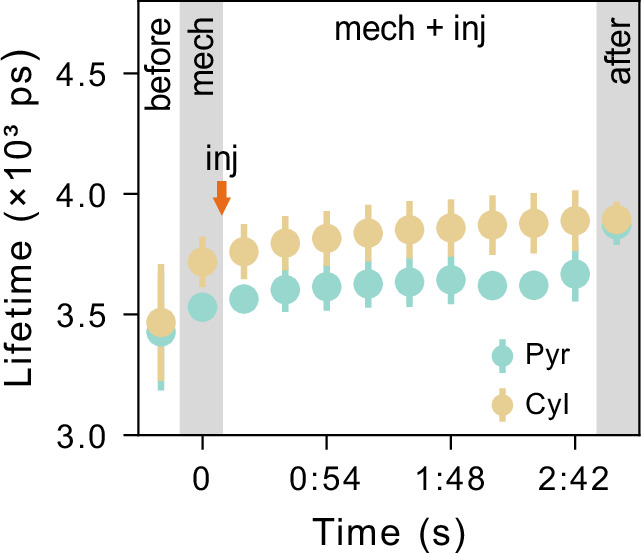
Lifetime values before, during, and after injecting HeLa cells with DMSO as control group with pyramidal and cylindrical probes at incident point (mean ± SD, *N* ≥ 3, *n* ≥ 11). Different sections on the graph represent lifetime measurements before stimulus, during mechanical stimulus when the probe has reached the set point on the cell without fluidic pressure application (‘mech’), and during mechano-chemical stimulus when the probe remains in contact with the cell at the set point, and a fluidic pressure pulse is applied for drug (or here control) injection (‘mech + inj’), and after retraction of probe and removal of the stimulus.

FluidFM probes were filled with 50 µM CytoD in DMSO (or DMSO for control groups) and used for mechano-chemical triggering by indenting the nucleus at 150 nN (‘mech’), followed by a quantitative intracellular injection of CytoD or DMSO (‘mech + inj’) (Figure 2A, B). The indentation force was adjusted to 150 nN to ensure a successful injection into HeLa cells. FLIM measurements were performed before, during ‘mech’ and ‘mech + inj’, and just after the manipulation (Figure 2B, C). Indentation force applied by AFM, serving as a controlled mechanical stimulus (black line), fluidic pressure pulse used for injection (orange line), and time-lapse lifetime values recorded by the FLIM detector (dark blue crosses and light blue fitted curve) were recorded simultaneously (Figure 2B). This injection process delivers ~10 fl of CytoD (or DMSO), equivalent to ~1% of the cell’s volume, leading to a final intracellular concentration of 0.5 µM. During ‘mech + inj’, the probe remained inside the cell for 3 min ±10 s. After this period, the probe was retracted, and the lifetime values of the cell were captured again. Next, we compared responses under mechano-chemically challenging conditions: injection of CytoD (Figure 2C) or DMSO (Figure 2—figure supplement 1). A markedly higher tension at the indentation site was observed for cylindrical probes compared to pyramidal ones. As observed before (Figure 1D), cylindrical probes exert a higher tension at the indentation site both with and without CytoD injection. In the case of pyramidal probes, tension increased at the indentation site at ‘mech’ state but remained constant for the entire ‘mech + inj’ state. In contrast, by cylindrical probes tension increases at the indentation site during the ‘mech’ state and increases with time after injection of CytoD (‘mech + inj’). After removal of the stimulus, tension at the indentation site shows an increase for pyramidal probes and a slight decrease for cylindrical probes, consistent with the results shown in Figure 1D.

Time-lapse lifetime images of representative cells were recorded at various stages: before, during mechanical stimulus (‘mech’), after mechanical stimulus and injection (‘mech + inj’), and post-retraction, using pyramidal and cylindrical probes (Figure 2D). Lifetime changes were calculated by subtracting each cell’s initial lifetime value and averaging these changes for each condition (CytoD or Cont), both during (Figure 2E) and after the stimulus (Figure 2F). CytoD-injected and control cells showed a similar tension across all the ROIs in the case of pyramidal probes (Figure 2E, top row). For cylindrical probes, CytoD-injected cells exhibited a slight increase (p-value of 0.087) at the ER and a significant increase (p-value of 0.015) at the NE when compared to control cells (Figure 2E, bottom row). Interestingly, after retracting probes (Figure 2F), tension at the ER and the NE remained higher for CytoD-treated cells compared to control groups when challenged by cylindrical probes. By retracting pyramidal probes, similar levels of tension were observed for CytoD- and control groups within all the ROIs. In agreement with Figure 1D, retracting pyramidal probes imposed an additional mechanical stress onto the cell (Figure 2E, F).

Elevated tension at the NE and indentation site is likely linked to actin filaments’ loss in CytoD-injected cells, as actin filaments are known to protect nuclei from excessive mechanical stress (Hoffman et al., 2020). Reportedly, external mechanical stimulation of cells, as well as intensive stretching of the NE during cell migration, triggers a transient formation of an actin rim around the nucleus or actin cap, which is hypothesized to protect nuclear content from mechanical damage by minimizing nuclear deformation (Fracchia et al., 2020). Therefore, hindering actin polymerization by CytoD injection inhibits actin cap formation, which otherwise dissipates the excessive strain, thus lowering the tension in response to external stimuli (Figure 2G).

Furthermore, the ER displayed increasing tension with time. As the NE is contiguous with the ER, stretching and deforming the nucleus is likely to increase the membrane tension in the neighboring ER (Enyedi and Niethammer, 2017). Additionally, the ER extends throughout the entire cell. Therefore, when targeting the nucleus, even with sharpened cylindrical probes, the ER is likely affected as well. However, similar tension at the ER and NE for CytoD-injected and control cells during and after stimuli was observed when pyramidal probes were used (Figure 2F). The pyramidal probes with a bigger apex size compared to cylindrical ones insert a mechanical disturbance directly to the ER and the NE while targeting the nucleus. This results in similarly elevated tensions for both control and CytoD-injected cells when stimulated with pyramidal probes.

Additionally, the interplay between actin dynamics and mechanotransmission at the ER and NE highlights the complexity of cellular mechanoresponses and emphasizes the need for further investigation into their cross-reactivity and directionality. Thus, we applied mechano-chemical triggering to a variety of target organelles, including the peripheral ER, nucleus, and cell periphery, and further studied the tension propagation in situ as a response to these stimuli.

### Interconnected mechano-crosstalk between ER, nucleus, and actinomyosin: investigating the impact of target organelle for the mechano-chemical stimuli

**Figure 3.**
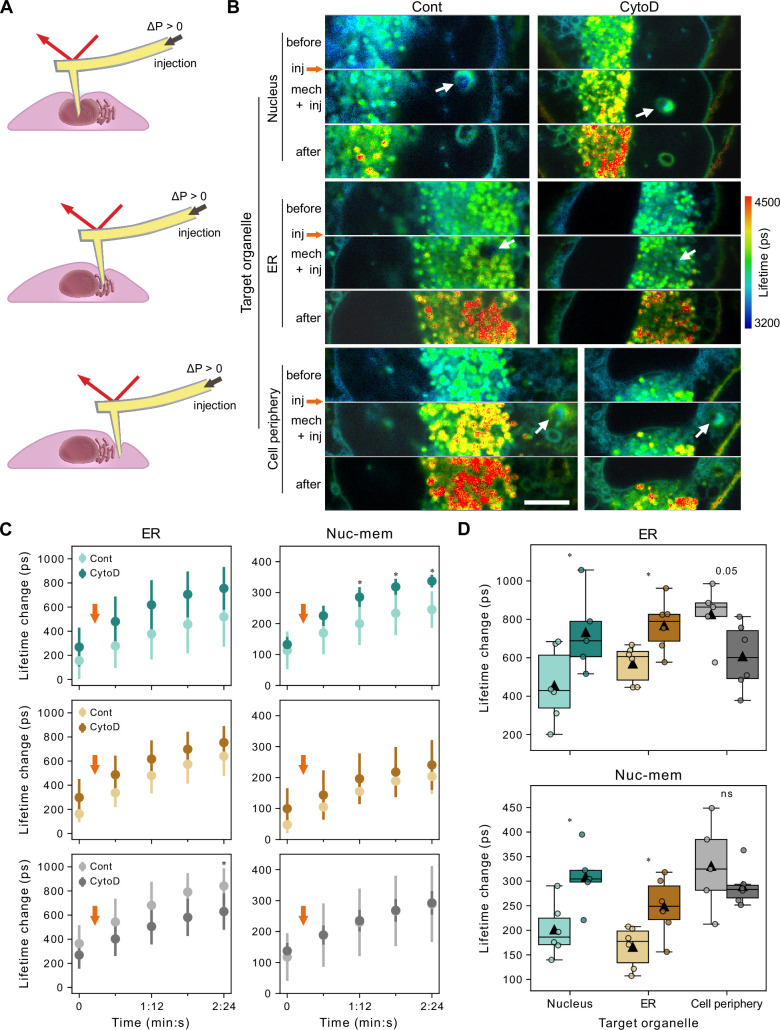
ER and nuclear envelope crosstalk as a response to mechano-chemical triggering of different intracellular components of HeLa cells. (**A**) Schematic shows targeting different organelles for mechano-chemical stimulation: nucleus (top), the ER (middle), and cell periphery (bottom). (**B**) Lifetime images of HeLa cells stained with the ER Flipper-TR before, at mechano-chemical stimulated state (‘mech + inj’), and after stimulation targeting different subcellular compartments: nucleus (top panel), the ER (middle panel), and cell periphery (bottom panel) for indentation and injection of either control (Cont) or CytoD using sharpened cylindrical probes. Arrows indicate where the cylindrical probe penetrates the cell (‘Indent-site’). Scale bar represents 5 µm. Change in lifetime at the ER and nucleus membrane (Nuc-mem) during (**C**, noted as ‘mech + inj’ in B) and after (**D**) stimuli imposing injection of DMSO as control (light) or CytoD (dark) at different subcellular compartments: nucleus (top panel), ER (middle panel), and cell periphery (bottom panel) (mean ± SD, *N* = 2, *n* ≥ 5, **p < 0.01, *p < 0.05, *t*-test). Orange arrows in C indicate the injection pulse at the specified time in the mechano-chemical process. Mean values are shown with triangles in boxplots in D (*N* = 2, *n* ≥ 5, *p < 0.05, reported p-values of 0.05 ≤ p < 0.1, ns: not significant, *t*-test).

The mechanical crosstalk between the ER, nucleus, and cytoskeleton is complex because of their intricate interconnection (Pruitt et al., 2014). While it is acknowledged that the nucleus serves as an endpoint for the dispersion of forces, there is limited research specifically addressing cross-reactivity and directionality of mechano-signaling between these subcellular components. Therefore, a cylindrical probe was used for indentation on either the peripheral ER, the nucleus (with minimal ER lumens above), or the cell periphery with limited ER involvement (Figure 3A, B). Besides, intracellular injection of either CytoD or DMSO (control) was performed. In parallel, the tension at the ER and the NE of cells was monitored during (Figure 3C) and after (Figure 3D) mechano-chemical triggering. When the nucleus was targeted (Figure 3C, top row), CytoD-injected cells showed higher tension at both the ER and NE compared to the control group. Conversely, for cell periphery-targeted cells (Figure 3C, bottom row), control cells showed a higher tension at both the ER and NE compared to CytoD-injected cells. Interestingly, when the ER was subjected to the same stimuli (Figure 3C, middle row), no detectable change was observed between the cells injected with or without CytoD. Following the probe retraction, tension at the ER and NE was higher for CytoD-injected compared to control cells when the ER and nucleus were targeted (Figure 3D). In contrast, in cells targeted for the cell periphery, slightly lower tension at the ER and similar tension at the NE were observed for CytoD-treated compared to control cells.

Higher tension at the ER for control cells compared to CytoD-injected cells suggests actin networks transmit strain and tension intracellularly, unlike intermediate filaments which absorb mechanical stress, preventing it from reaching the nucleus (Oses et al., 2023). Therefore, interrupting actin filaments likely keeps tension localized at the indentation site. This is validated by higher tension transfer to the ER in control cells compared to CytoD-treated ones when the cell periphery is targeted (Figure 3C, top and bottom rows). The ER’s complex morphology consists of many curved structures of lumens and disks which can deform when subjected to external mechanical perturbation, making it prone to absorb stress and strain when directly targeted. That could explain the similar tension levels in both CytoD-injected and control cells during ER indentation. Notably, unlike nucleus-targeted cells, ER-targeted cells only show increased tension at the ER and NE in CytoD-injected cells compared to control ones after stimulation. This suggests fundamental differences in the mechanical coupling of the nucleus and the ER to the cytoskeleton. While the nucleus maintains direct, structural actin connections through the nuclear lamina and LINC complexes (Maurer and Lammerding, 2019), making it immediately sensitive to actin disruption, the ER relies on indirect, signaling-mediated cytoskeletal interactions (Shi et al., 2022; van Vliet and Agostinis, 2017). Thus, the ER functions as a dynamic tension buffer that engages cytoskeletal support primarily during active repair processes following mechanical perturbation. This explains why nuclear probing reveals immediate tension differences in actin-disrupted cells, while ER probing only shows post-retraction effects. Consequently, statistical analysis detects significant differences between test and control groups after probe removal, but not during probe contact in ER-targeted experiments. Yet, when the nucleus is targeted, higher tension at both the NE and the ER is recorded for CytoD-treated compared to control cells both during and after the stimuli. Such distinct mechanoresponse patterns from the ER and the NE (Figure 3C, top and middle rows) also hint at a directional mechano-crosstalk between the ER and the nucleus of a stimulated cell.

Nuclear lamins regulate nuclear mechanoresponse. Therefore, we investigated how different lamins impact mechano-signaling in HeLa cells upon mechano-chemical stimulation of the nucleus, to shed light on dynamic tension responses.

### Lamin A/C’s autonomous contribution to nucleus mechanoresponsiveness and its dependence on B-type lamins only beyond a sustained force threshold

The nuclear lamina regulates nuclear tension and contributes to cellular mechanotransmission (Guilluy et al., 2014). Lamin A/C modulates nuclear stiffness and chromatin remodeling (Donnaloja et al., 2020), while its spatial distribution determines nuclear deformability (Srivastava et al., 2021). B-type lamins have little effect on nuclear stiffness (Srivastava et al., 2021) but maintain NE integrity (Camps et al., 2015). Moreover, a balance between lamin A/C and lamins B1 and B2 is important for nuclear elasticity, with an increased ratio of lamin A/C to the B-type lamins corresponding to a stiffer nucleus. The interplay between A- and B-type lamins, their post-translational modifications, and their impact on governing the nuclear deformability thus genome organization remain underexplored (Cho et al., 2019). Here, we examined the contribution of nuclear lamins to changes in membrane tension using HeLa cells with altered nuclear lamina composition upon downregulation of A- and/or B-type lamins.

**Figure 4.**
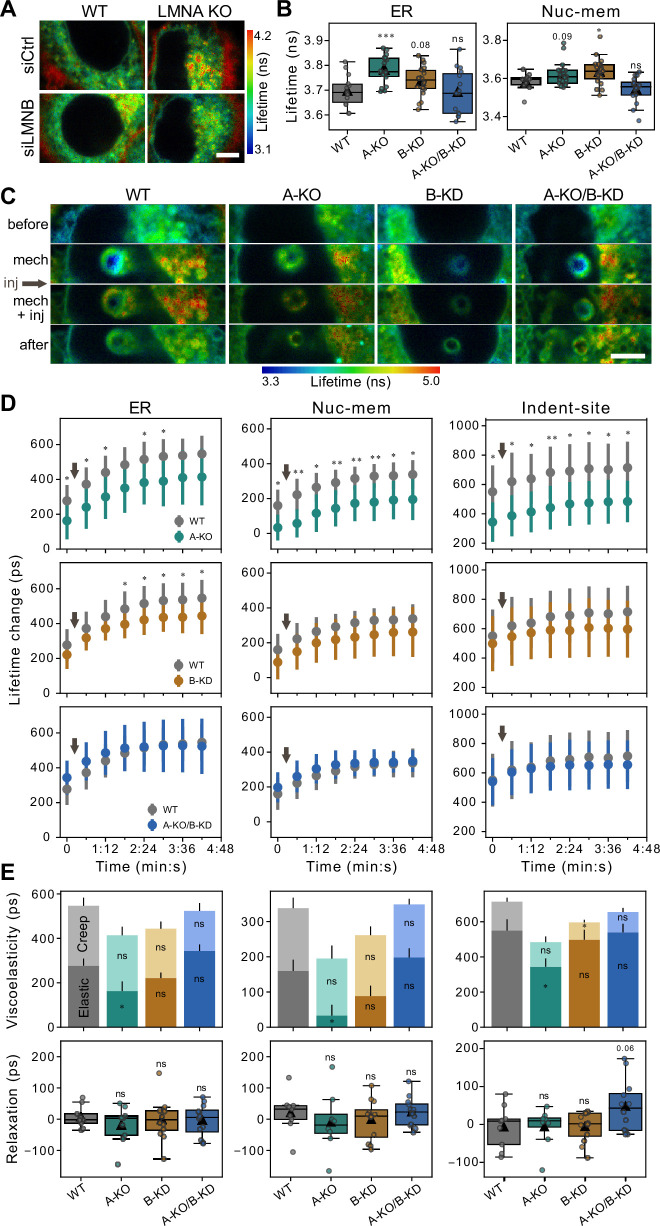
A-type lamins contribute to elasticity, while B-type lamins may influence viscosity in the nucleus’s viscoelastic behavior under sustained deformation with disrupted actin polymerization. Lifetime images (**A**) and boxplots showing the absolute lifetime values (**B**) of HeLa cells with varied levels of downregulation of lamin proteins: WT-siCtrl (WT), WT-siLMNB (A-KO), LMNA KO-siLMNB (B-KD), and LMNA KO-siLMNB (A-KO/B-KD). Cells were stained with ER Flipper-TR dye. Scale bar in A represents 5 µm. Mean values are shown with triangles in B (*N* = 2, *n* ≥ 8, ***p < 0.001, *p < 0.05, reported p-values of 0.05 ≤ p < 0.1, ns: not significant, *t*-test). (**C**) Lifetime images of WT, A-KO, B-KD, and A-KO/B-KD cells stained with ER Flipper-TR before, upon indentation (‘mech’), upon indentation + CytoD injection, ‘mech + inj‘, and after stimulation using sharpened cylindrical probes. Note the extended range for lifetime, compared to A, revealing distinguished cell responses to the stimuli, thus indicating the initial tension state for different cell versions in the same color. Scale bar represents 5 µm. (**D**) Lifetime change at the ER, nucleus membrane (Nuc-mem), and indentation site during mechano-chemical stimuli. All cells were injected with CytoD. In each panel, the tension response of WT cells was compared with either of A-KO, B-KD, and A-KO/B-KD cells. Orange arrows indicate the injection pulse at the specified time in the mechano-chemical process. Time zero corresponds to the indented state (indicated by ‘mech’ in C) which follows by an injection (indicated by ‘mech + inj’ in C) (mean ± SD, *N* = 3, *n* ≥ 9). (**E**, top) Viscoelastic response of cell compartments (left to right: ER, Nuc-mem, and Indent-site) with varied depletion of lamins to the mechano-chemical stimuli composed of lifetime change originating from the instantaneous cell response (Elastic) upon indentation in nucleus (first time points in D) and from the time-dependent response (Creep) due to atomic force microscope (AFM) probe being in contact with the nucleus (at 150 nN) for around 4 min after CytoD injection (last time points in D). Data represent the mean ± SEM (*N* = 3, *n* ≥ 9, ***p < 0.001, **p < 0.01, *p < 0.05, reported p-values of 0.05 ≤ p < 0.1, *t*-test). (**E**, bottom) Relaxation behavior of WT, A-KO, B-KD, and A-KO/B-KD cells at varied positions (left to right: ER, Nuc-mem, and Indent-site) after stimuli reported as lifetime change right after probe retraction compared to last time point during stimuli (last time points in D). Mean values are shown with triangles in boxplots (*N* = 3, *n* ≥ 9, reported p-values of 0.05 ≤ p < 0.1, ns: not significant, *t*-test).

First, WT and *LMNA* KO HeLa cells were subjected to RNA interference (RNAi), using a non-targeting silencing RNA (siRNA) control pool (siCtrl) or pools of siRNAs targeting lamin B1 and lamin B2 (siLMNB) for simultaneous KD of both B-type lamins. The use of RNAi yielded 4 groups of conditions: WT-siCtrl (WT), *LMNA* KO-siCtrl (A-KO), WT-siLMNB (B-KD), and *LMNA* KO-siLMNB (A-KO/B-KD). Western blot analysis confirmed successful depletion of corresponding lamins in each group (Figure 4—figure supplement 1). Cells were then stained with the ER Flipper-TR dye and assessed for changes in tension levels (Figure 4A). Lifetime values at the ER and NE were quantified and compared for the different groups (WT, A-KO, B-KD, and A-KO/B-KD; Figure 4B). Higher tension was captured at the ER and NE for both A-KO and B-KD compared to WT cells. A-KO cells displayed significantly higher tension at the ER compared to WT cells, whereas B-KD cells showed a slightly elevated tension level in comparison to WT. Surprisingly, the A-KO/B-KD group showed no captured changes in tension at either the ER or NE in comparison to WT cells. Next, we indented cells by cylindrical probes followed by CytoD injection (Figure 4C), and quantified tension level in the same manner as before (Figure 4D). A significantly lower tension at the ER, NE, and indentation site for A-KO compared to WT cells was detected. Intriguingly for B-KD cells, similar tension levels were observed at both the NE and the indentation site when compared to WT cells. At the ER though, like A-KO cells, B-KD cells showed lower tension in response to external stimulus when compared to WT cells. Surprisingly, no changes in tension level of A-KO/B-KD cells were detected compared to WT cells. As varying lamin compositions may alter nuclear stiffness, we also measured indentation depth under a constant 150 nN force (Figure 4—figure supplement 3). We found no statistically significant difference in the indentation depth between groups. The differing tension responses are therefore due to intrinsic differences in molecular mechanics under identical stimulus. Examining the kinetic traits of WT and A-KO/B-KD cells revealed a reduced slope in the tension response from cells lacking A- and B-type lamins, particularly noticeable in the steady-state response at 4 min post-stimulus (Figure 4—figure supplement 4).

**Figure 4—figure supplement 1.**
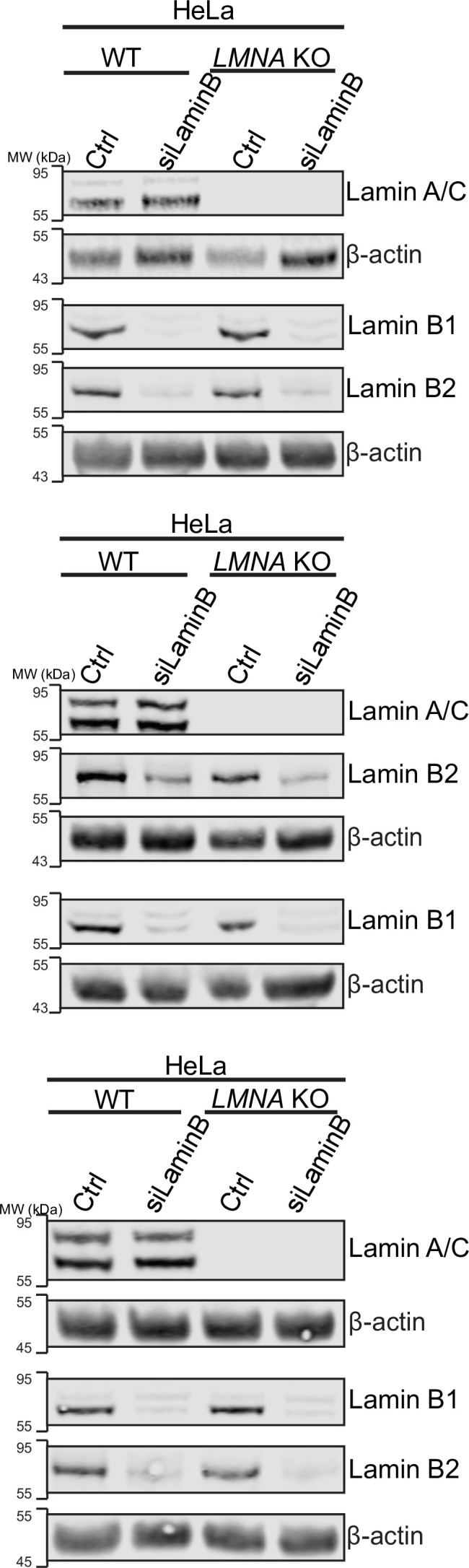
Western blot analysis verifying the absence of lamin A/C in *LMNA* KO cells and the successful downregulation of lamins B1 and B2 in both WT and *LMNA* KO upon treatment with siLMNB. Three biological replicates were utilized for mechano-chemical triggering assays and FLIM-based tension measurements. Figure 4—figure supplement 1—source data 1.PDF file showing the unprocessed gels used in the presented blots in Figure 4—figure supplement 1. Figure 4—figure supplement 1—source data 2.ZIP file containing the unprocessed gels of the three western blot replicates shown in Figure 4—figure supplement 1.

**Figure 4—figure supplement 2.**
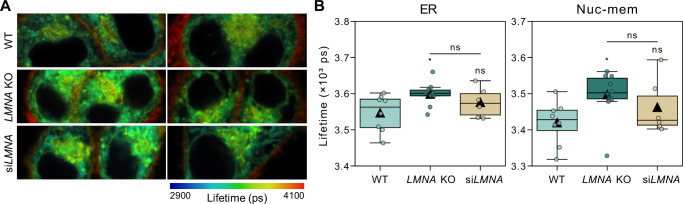
Lifetime images (**A**) and boxplots showing absolute lifetime values at the ER and nuclear membrane (Nuc-mem) (**B**) of HeLa cells stained with ER Flipper-TR tension reporter molecule. The lamin A/C level of cells was downregulated with altering methods: WT, *LMNA* KO, and WT siLMNA. Cells. Scale bar in A represents 5 µm. Mean values are shown with triangles in boxplots in B (*N* = 2, *n* ≥ 6, *p < 0.05, ns: not significant, *t*-test with Holm correction).

**Figure 4—figure supplement 3.**
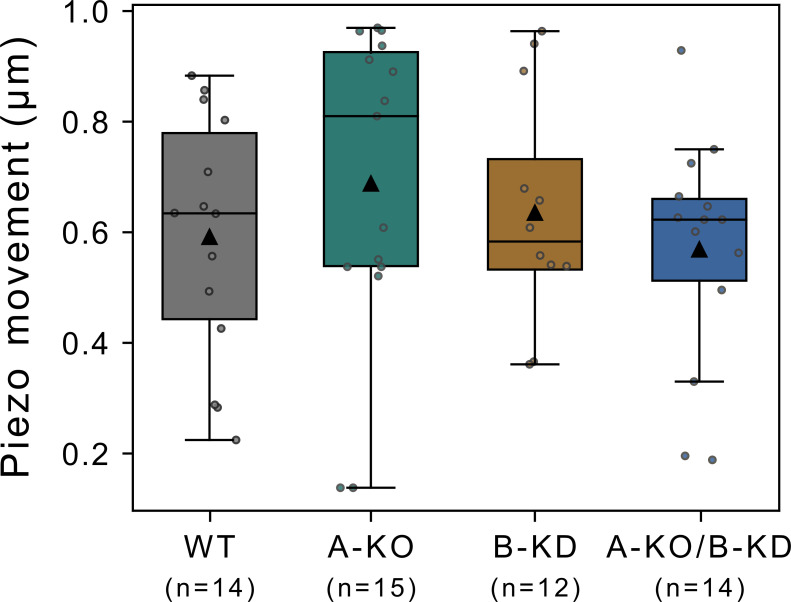
Relative piezo movement from the contact point until reaching 150 nN setpoint for cells with varied lamina compositions: WT, A-KO, B-KO, and A-KO/B-KD. Mean values are shown with triangles in boxplots (*N* = 3, *n* ≥ 12 cells). No statistically significant differences were observed between any groups (Kruskal–Wallis *H*-test: *H* = 1.744, p = 0.627, ns: not significant, Mann–Whitney *U* tests with Bonferroni correction).

**Figure 4—figure supplement 4.**
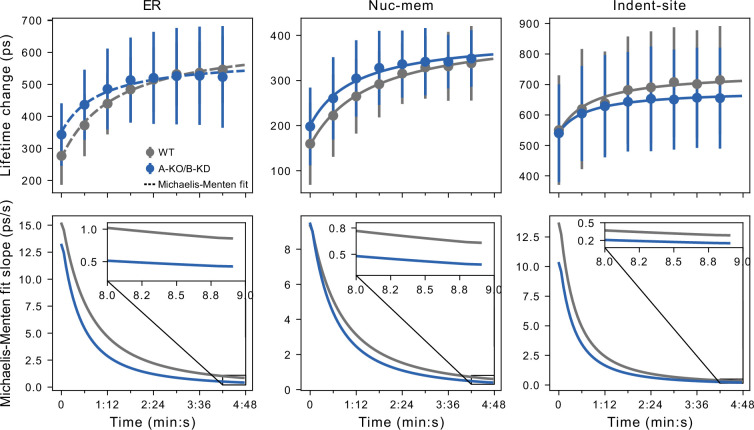
Michaelis–Menten fit to lifetime response (top) and slope value of fitted curve (bottom) in HeLa cells with and without A- and B-type lamins at the ER, nuclear membrane (Nuc-mem), and indentation site (Indent-site) (mean ± SD, *N* = 3, *n* ≥ 9).

**Figure 4—figure supplement 5.**
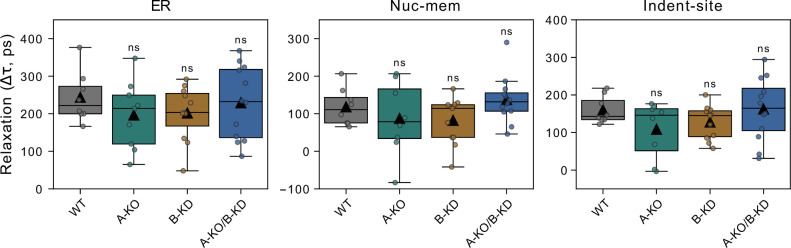
Relaxation behavior of cell with different levels of lamin proteins: WT, A-KO, B-KO, and A-KO/B-KD at the ER, nuclear membrane (Nuc-mem), and indentation site (Indent-site) after stimulus. Relaxation is reported as lifetime change 5 min after probe retraction compared to last time point of stimuli. Mean values are shown with triangles in boxplots (*N* = 3, *n* ≥ 9, ns: not significant, *t*-test).

These results align with earlier findings stating that cells lacking lamin A/C, but not lamins B1 and B2, have softer nuclei with a more deformable NE, thus dissipating the stress and lowering the tension at the NE (Williamson et al., 2018; Solovei et al., 2013). This leads to a lower tension level at the NE for A-KO cells compared to WT cells when exposed to external stimuli. The elevated wrinkles in the NE of lamin A/C KO cells (Kim et al., 2017) might play a role in dissipating stress and reducing tension during mechanical stimuli, particularly when these wrinkles open due to the deformation. In essence, a nucleus without A-type lamins appears less mechanoresponsive, as it likely absorbs forces through bending rather than transferring them. In agreement with the previous studies, B-type lamins, on the other hand, do not affect nuclear stiffness and deformation, thus its tension response.

Lower tension in A-KO and B-KD cells compared to WT cells suggests that both A- and B-type lamins influence the tension crosstalk from the NE to the ER. Examining their tension response reveals that when actin filaments are disturbed, lamin A/C primarily affects the immediate response of the nucleus to physical deformation of the nucleus. Conversely, B-type lamins become more significant under sustained load. In other words, lamin A/C alone can contribute to the nucleus’s mechanoresponsive abilities, but *LMNA* KO cells become less mechanoresponsive than WT cells and depend on the supportive function of B-type lamins. This implies that even at this juncture, A-KO/B-KD cells under prolonged stress might show reduced mechanoresponses compared to WT cells, notably with a decrease in tension at the indentation site recorded after 3 min post-stimulus (Figure 4D).

The nucleus stretches rapidly in response to a step-function load and undergoes a viscoelastic transition with sustained load, taking seconds to reach a steady state. This behavior depends on lamina composition and chromatin states (Buxboim et al., 2023), possibly aiming to dissipate mechanical energy and prevent nuclear damage. We discerned the different roles of lamin A/C versus lamin B1 and B2 in the viscoelastic response of the nucleus and the ER. This process separated the immediate response (‘elastic’), due to the probe indentation measured at ‘mech’ state from a gradual deformation (‘creep’) under constant force applied during ‘mech + inj’ state. Creep response reflects the cell’s viscous property, with higher creep reflecting lower cell’s viscosity. The viscoelastic response was influenced by CytoD injection as a chemical stimulus, followed by a continuous load of 150 nN for 4 min ±10 s. The results are depicted as accumulated bar plots in the top panel of Figure 4E. At the ER, similar levels of elastic and viscous responses were detected for cells with different lamin proteins levels compared to WT cells. At the NE, though, lower elasticity in *LMNA* KO cells and similar viscous response in all the cells were captured. At the indentation site, cells lacking A-type lamins show a reduced elastic property while cells with deficiencies in B-type lamins have lower creep and thus higher viscous response. Further, the temporal relaxation of tension at the ER, NE, and indentation site were investigated both immediately after the stimulus (Figure 4E, bottom row) and 5 min post-stimulus (Figure 4—figure supplement 5). Relaxation property was quantified by comparing the lifetime value after probe retraction to the last time point of stimuli at ‘mech + inj’ state. A higher relaxation value indicates greater recovery of the deformed structure and thus a stronger elastic component of the mechanoresponse. Following the stimulus, cells deficient in both A- and B-type lamins displayed increased relaxation at the indentation site which experiences maximum deformation (Figure 4E, bottom right). Finally, 5 min post-stimulus, cells with diverse depletion of lamins exhibit a consistent relaxation extent across various ROIs (Figure 4—figure supplement 5). These findings suggest A-type lamins contribute to nuclear elasticity, while B-type lamins influence viscous behavior during sustained deformation and disrupted actin polymerization (Figure 4E, top right). Nonetheless, previously a potential role for lamin A in the viscous response of NE in both intact tissue and suspended cells has been reported (Swift et al., 2013; Harada et al., 2014). In our study, the disruption of the dynamic actin network by CytoD injection suggests that the interaction between lamin A/C and the actin meshwork may be essential for maintaining the viscous properties of lamin A. Higher relaxation in A-KO/B-KO cells immediately upon removal of probes, reflecting higher tension restoration, may be linked to the absence of actin filaments alongside the lamins depletion. In contrast, WT cells and cells lacking either A- or B-type lamins maintain tension, releasing it gradually over time. Similar relaxation values for all cells at 5 min post-stimulus indicate cytoskeletal reorganization and NE adaptations, allowing stress recovery independently of nuclear lamina. Since nuclear lamina organization affects how cells detect and respond to mechanical stimuli, we aimed to dissect the roles of cytoskeletal components in initiating the mechanoresponse of the nucleus and ER after mechanical and chemical stimuli with various molecular disruptors (CytoD, Noco, and their combination).

### Modulating mechanoresponse: impact of lamin A/C on MT dynamics and adaptive mechanosensitivity

The nuclear lamina contributes to organize the cytoskeleton (Oses et al., 2023), with other components like actin filaments and MTs enabling cells to sense and respond to mechanical stimuli (Figure 2G; Liu et al., 2020). In particular, loss of lamin A/C is linked to decreased myosin II contractility and in some cases to the absence of apical stress fibers (Alisafaei et al., 2019), which disrupts cytoskeletal dynamics and affects how cells interpret mechanical signals. Conversely, MT depolymerization can enhance actomyosin contractility via the RhoA–Rock pathway, altering cell mechanics and nuclear properties. Considering the intricate interplay between the cytoskeleton, nucleus, and mechanical cues in cellular behavior, we assessed how changes in cytoskeletal components and lamin A/C affect cellular mechanoresponse and disease contribution.

Noco- and CytoD-Noco-treated WT cells had low tension across ROIs. A-KO cells showed higher tension at the ER and NE with Noco or CytoD-Noco compared to CytoD alone. No difference in tension between Noco and CytoD-Noco groups at the ER and NE.

**Figure 5.**
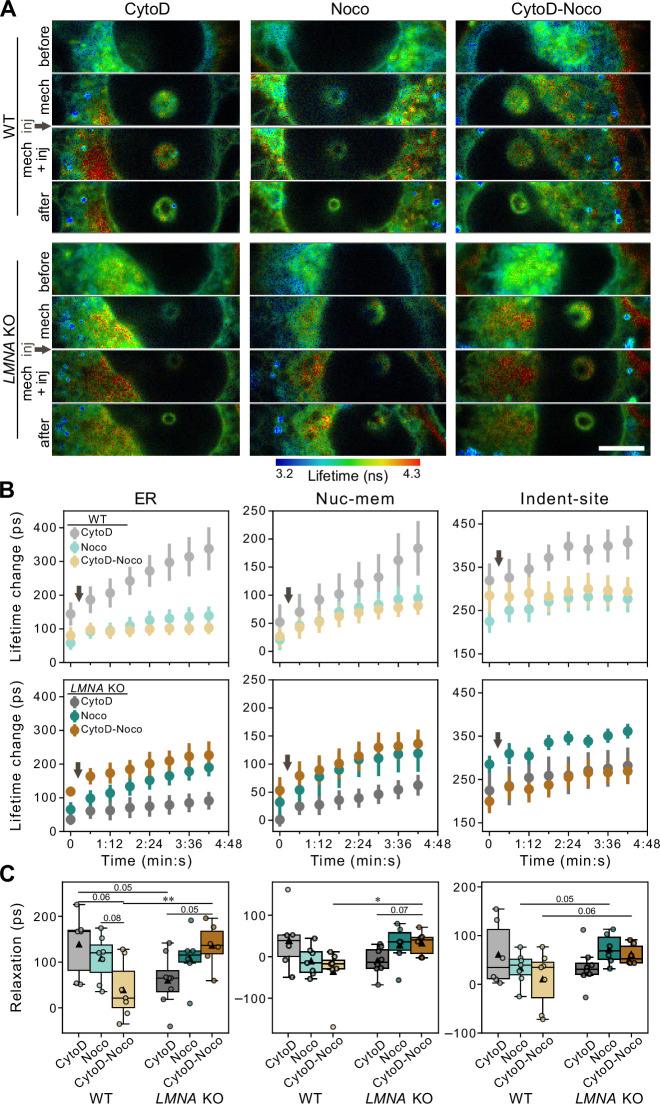
Dynamic role of microtubules and actinomyosin in regulating mechanoresponse of nuclei in cells with and without lamin A/C depletion to the external stimulus. (**A**) Lifetime images of WT- and LMNA KO-HeLa cells stained with ER Flipper-TR before, upon indentation (‘mech’), upon indentation + injection (‘mech + inj’) of various drugs and after stimulation using sharpened cylindrical probes. For chemical stimulation, interruption of different cytoskeleton components including actin filaments, microtubules, and both actin filaments and microtubules was targeted realized by injection of 50 µM CytoD (left column), 50 µM Noco (middle column), and 50 µM CytoD + 50 µM Noco (right column) dissolved in DMSO. Scale bar represents 5 µm. (**B**) Lifetime change at ER, nucleus membrane (Nuc-mem), and indentation site during stimuli in WT and LMNA KO cells. Arrows indicate the injection pulse at the specified time in the mechano-chemical process. Time zero corresponds to the indented state (indicated by ‘mech’ in A) which follows by injection, indicated by ‘mech + inj’ in A (mean ± SEM, *N* = 2, *n* ≥ 6, **p < 0.01, *p < 0.05, reported p-values of 0.05 ≤ p < 0.1, *t*-test with Holm correction). (**C**) Relaxation behavior of WT and LMNA KO cells at varied positions (left to right: ER, Nuc-mem, and Indent-site) 5 min after mechano-chemical stimuli involving various drugs (CytoD, Noco, and CytoD-Noco). Relaxation is reported as lifetime change at 5 min after probe retraction compared to the last time point of stimuli (last time points in B). Mean values are shown with triangles in boxplots (*N* = 2, *n* ≥ 6, **p < 0.01, *p < 0.05, reported p-values of 0.05 ≤p < 0.1, *t*-test with Holm correction).

Actomyosin and MTs, due to their dynamic response compared to intermediate filaments (Geng et al., 2023), were studied for their roles in initiating nuclear mechanoresponse. We targeted the cytoskeleton of WT and A-KO cells with different disruptors including CytoD, Nocodazole (Noco, a MT inhibitor), and their combination (CytoD-Noco), each at 50 µM, resulting in 0.5 µM upon intracellular injection. Noco binds to β-tubulin subunits, thereby inhibiting the polymerization of tubulins into MTs (Blajeski et al., 2002). These investigations focused on WT and lamin A/C-deficient cells, due to lamin A/C’s distinctive roles in NE mechanoresponse as examined in section 2.4. Tension response to stimuli was quantified at various ROIs (Figure 5A, B). Notably, WT cells’ mechanoresponse is governed by MTs’ presence, showing increased tension in CytoD-treated cells compared to Noco- or CytoD-Noco-treated cells at the ER, NE, and the indentation site. Tension in WT cells with MTs (CytoD-treated cells) compared to those with less MTs (either Noco- or CytoD-Noco-treated cells) significantly increased upon stimulation. Noco- and CytoD-Noco-treated WT cells exhibited similarly low levels of tension across all ROIs. Cells lacking lamin A/C showed distinct responses to the aforementioned drugs compared to WT cells. *LMNA* KO cells displayed higher tension at the ER and NE when subjected to Noco or CytoD-Noco (less MTs in function) compared to when exposed to CytoD alone. Interestingly, at the ER and NE there were no differences in tension values between Noco and CytoD-Noco groups. At the indentation site, A-KO cells showed similar trends in the ER and NE for CytoD- and Noco-treated cells. Interestingly, A-KO cells lacking both actin filaments and MTs had the lowest tension.

**Figure 5—figure supplement 1.**
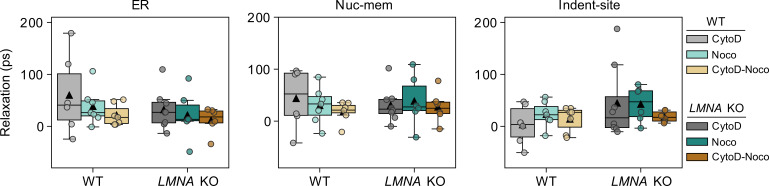
Relaxation behavior of WT and *LMNA* KO cells immediately after mechano-chemical stimuli involving various drugs (CytoD, Noco, and CytoD-Noco), at the ER, nuclear membrane (Nuc-mem), and indentation site (Indent-site). Relaxation is reported as lifetime change following the retraction of the probe compared to the last time point of stimuli. Mean values are shown with triangles in boxplots (*N* ≥ 2, *n* ≥ 6, no significant differences were captured from *t*-test with Holm correction).

Higher tension in WT cells with active MTs compared to those with less MTs (Noco- and CytoD-Noco-treated) indicates MTs’ dominant role in tension propagation within the ER–NE network. This is likely due to their close association with the ER network. MTs seemingly maintain tension within the ER–NE network rather than transferring it, independently of actin filaments (Figure 5B, top row). Yet, actin filaments lower the tension in cells with intact MTs (Figure 2E, F). In A-KO cells, MTs facilitate nuclear deformation (Tariq et al., 2017). Thus, in A-KO cells, Noco and CytoD-Noco treatment minimizes the deformation of the nucleus and increases tension at the ER and NE. On the other hand, CytoD-treated A-KO cells with functional MTs exhibit higher deformation and thus lower tension. Similar tension levels in Noco- and CytoD-Noco-treated A-KO cells at the ER and NE indicate actin filaments independence. Lamin A/C forms the perinuclear actin cap (Kim et al., 2017; Kechagia and Roca-Cusachs, 2023), explaining high tension at the indentation site in Noco-treated A-KO cells compared to WT cells despite having active actin filaments. Low tension in CytoD-Noco-injected A-KO cells suggests a highly deformable nuclear membrane, despite lacking active MTs at the indentation site.

Finally, temporal tension relaxation was studied upon probe removal and diminishing the mechanical stimulus. Relaxation was quantified as a change in lifetime immediately after probe retraction (Figure 5—figure supplement 1) and 5 min (Figure 5C) post-stimulus, both with respect to the last time point during stimuli. WT and lamin A/C-deficient cells exhibited similar tension immediately after probe retraction, regardless of the injected drug. Additionally, lamin A/C-deficient cells treated with various drugs had consistent relaxation compared to WT across all ROIs. Though, relaxation at 5 min post-stimulus depended on drug treatment, especially at the ER and NE. WT cells' treatment with CytoD or Noco alone had higher relaxation compared to CytoD-Noco at the ER. Instead, A-KO cells showed the lowest relaxation with CytoD and highest with CytoD-Noco treatment, 5 min post stimulus at the ER and NE. Notably, no significant differences in relaxation were found among drug treatments at the indentation site for both WT and *LMNA* KO cells.

Collectively, both actin and MTs contribute to the cell’s elastic mechanoresponse, as shown by the similar elastic relaxation in WT cells treated with CytoD or Noco. Interestingly, loss of both A- and B-type lamins does not affect tension at the NE or ER, unlike the individual loss of either lamin type. This highlights the complex interplay between different lamins and cytoskeletal elements in cellular mechanics. MTs play a lesser role in conferring viscous properties (time-dependent) to healthy cells, indicated by lower creep and higher viscous response of the ER when exposed to MT-disturbing drugs (Noco or CytoD-Noco). This is due to the rapid dynamics of MTs with critical roles in cell division and intracellular transport (Akhmanova and Steinmetz, 2010; Schaedel et al., 2015). In lamin A/C-deficient cells, MTs contribute to a more viscous mechanoresponse, shown by higher creep and lower viscous response with MT-disturbing drugs (Noco or CytoD-Noco). This adaptation in MT mechanosensitivity may be due to the absence of the actin cap in lamin A/C-depleted cells.

### Conclusion

Combining FluidFM with FLIM enabled real-time, high-resolution mapping of intracellular tension dynamics upon quantitative injection of active components while maintaining a constant mechanical stimulus. The proposed approach allowed investigation of intracellular mechanotransmission crucial for mechanotransduction regulation and cell fate control. CytoD injection, as an in situ mechano-chemical trigger, disrupted actin filament dynamics, altering tension at the ER and NE. Sharpened cylindrical probes increased tension locally on the nucleus, and CytoD-injection increased tension at the ER and NE compared to control injection. Pyramidal probes required a higher force to puncture the nuclear membrane (50 nM) compared to that of the cylindrical one (10 nN). Membrane puncture resulted in tension relaxation at the NE. Increased indentation force for sealed injection showed lower tension at the NE and the ER with cylindrical probes compared to pyramidal ones. Changing the incident site from the nucleus to the ER and cell periphery confirmed that actin filaments alone transmit tension away from the deformation site. In contrast, CytoD injection and actin filament disturbance retained tension at the site of incidence. Direct ER targeting suppressed tension from reaching the nucleus. This indicates specialized tension distribution within cells. Nuclear lamins showed distinct responses to mechanical cues, with A- and B-type lamins affecting elastic and viscous properties, respectively. Interestingly, combined loss of lamins A and B did not affect tension at the NE or ER, suggesting a compensatory mechanism needing further investigation.

In conclusion, our study examined how the cytoskeleton, particularly actin and MTs, collaborates with nuclear lamina to initiate nuclear mechanoresponse. In WT cells, MTs maintained tension, while actin filaments lowered it in responding to external stress. In lamin A/C-deficient cells, MTs facilitated nuclear deformation and lowered the tension, in contrast to actin filaments. This highlights lamin A/C’s role in regulating the collaboration between the NE and the cytoskeleton, affecting nuclear and the ER mechanoresponse. Additionally, nuclear deformation under external stimulus involves interactions between chromatin and cytoskeletal components through the lamina and LINC complexes. However, the exact mechanisms of chromatin involvement require further study. Lastly, the intermediate filament system, particularly vimentin, also plays a key role in nuclear integrity and connecting to other organelles like mitochondria. Future studies should explore their contributions to mechanotransmission phenomena.

## Materials and methods

### Materials

DMEM (Dulbecco’s Modified Eagles Medium + GlutaMAX, Thermo Fisher Scientific, Waltham, USA), CO_2_ Independent Medium (Thermo Fisher Scientific, Waltham, USA), FBS (fetal bovine serum, Thermo Fisher Scientific, Waltham, USA), penicillin–streptomycin (Thermo Fisher Scientific, Waltham, USA), Trypsin (Thermo Fisher Scientific, Waltham, USA), PBS (phosphate-buffered saline, Thermo Fisher Scientific, Waltham, USA), coumarin 120 (7-amino-4-methylcoumarin, Sigma-Aldrich, St. Louis, USA), SL2 Sigmacote (Sigma-Aldrich, St. Louis, USA), ER Flipper-TR (LubioScience, Zurich, Switzerland), and NucBlue (Thermo Fisher Scientific, Waltham, USA).

### Cells preparation

HeLa cells were chosen as a model system for mechanoresponsive studies due to the availability of genetically modified versions with altered levels of lamin proteins. HeLa cells were cultured at 37°C and 5% CO₂ in DMEM culture medium supplemented with 10% FBS and 1% penicillin–streptomycin using standard procedure for adherent cells. Cells were regularly tested for mycoplasma contamination by PCR. For splitting, 0.05% trypsin was used. Cells were seeded on glass bottom dishes (Willco, Amsterdam, Netherlands) with a seeding density of 6000 or 4000 cells/ cm^2^, 1 or 2 days before the experiments. Just before the experiments, cells’ medium was changed to CO_2_ independent medium and cells were stained with 1 µM ER Flipper-TR, designed to report changes in membrane tension at the ER. However, ER Flipper-TR dye apparently stains other endomembrane structures in addition to ER as well.

### Generation of lamin KO and KD cells

HeLa lamin A gene (*LMNA*) KO cells have been described previously (Luithle et al., 2020). RNAi-mediated depletion of B-type lamins was accomplished by siPOOLs (siTOOLs Biotech). Wild-type HeLa (WT) or HeLa *LMNA* KO cells were seeded into 6-well plates and transfected with 5 nM small interfering RNA (siRNA) pool targeting each protein of interest, or a pool of non-targeting control siRNAs using the transfection reagent RNAiMAX (Thermo Fisher Scientific, Waltham, USA). After 72 hr, cells were harvested for western blots or subjected to microscopic analysis. Additionally, to rule out clonal anomalies in the *LMNA* KO cells, HeLa WT cells were knocked down for lamin A/C, using a 5 nM pool of siRNA. Subsequently, these *LMNA* KD cells, *LMNA* KO cells, and WT cells were stained with ER Flipper-TR, and their initial lifetime time values were quantified and compared (Figure 4—figure supplement 3).

### Immunoblotting

Harvested cell pellets were lysed in SDS sample buffer (75 mM Tris pH 7.8, 20% (vol/vol) glycerol, 4% (wt/vol) SDS, 50 mM DTT, 0.01% (wt/vol) bromophenol blue) to yield whole cell lysates. Proteins were separated on pre-cast SDS–PAGE gels (vendor) and then transferred to a nitrocellulose membrane (GE Healthcare) using semi-dry blotting. The membrane was then blocked using blocking solution (5% milk in PBST) for 30 min, before being incubated with primary antibody diluted in blocking solution overnight at 4°C. The membrane was washed thrice for 5 min using blocking solution and subsequently incubated with secondary antibody diluted in blocking solution. Finally, the membrane was washed three times using PBST. Membranes were then developed using a LI-COR (Biosciences) to probe for a protein of interest.

### FluidFM probe preparation

As detailed previously (Gäbelein et al., 2022), cylindrical probes were custom-fabricated (SmartTip BV, Enschede, the Netherlands) featuring a tube length of 10 µm, an aperture diameter of 1 µm, and a microchannel thickness of 1.5 µm. Cylindrical probes were affixed to a custom-made probe holder for carbon coating (18 nm) using a CCU-010 Carbon Coater (Safematic, Switzerland) before milling with an FIB machine mounted on a SEM (Helios 5 UX, ScopeM, ETH Zurich, Switzerland) to achieve sharpness at their apex at a 50° angle (Figure 1B). FIB-milled cylindrical probes were then glued onto a cytoclip holder by Cytosurge (Cytosurge AG, Switzerland). Pyramidal probes (Nanosyringes, Cytosurge AG, Opfikon, Switzerland) and cylindrical probes were both subjected to oxygen plasma treatment (100-E Plasma System, Technics Plasma GmbH, Munich, Germany) for 2 min. Following this, they underwent an overnight coating with Sigmacote vapor inside a vacuum-sealed glass desiccator. This coating imparts anti-fouling properties to the probes, minimizing contamination by cell debris during experiments. Subsequently, the probes were baked at 100°C for 2 hr to ensure a stable coating. They were then stored alongside humidity-absorbing salt (Drierite, calcium sulfate, Sigma-Aldrich, St. Louis, USA) and utilized within 3 days post-coating. Prior to conducting the experiments, the spring constant of empty probes, initially designated with a nominal value of 1 N m^–1^, was determined using optical beam deflection and employing the Sader method (Bonyár et al., 2024; Nagy et al., 2019). Subsequently, the deflection sensitivity of the probes, filled with 0.1 mg ml^–1^ coumarin 120 (blue dye) plus the drug of interest for intracellular injection (50 µM cytochalasin D (CytoD), 50 µM nocodazole (Noco), or 50 µM CytoD + 50 µM Noco) in HEPES2 buffer, was measured.

### FluidFM-FLIM setup

As detailed previously (Lüchtefeld et al., 2024), FluidFM setup for micromanipulation of cells combined with FLIM imaging setup on the Leica SP8 FALCON inverted confocal microscope (Leica Microsystems GmbH, Wetzlar, Germany). To facilitate this, the condenser lens and z-galvo stage of the confocal microscope were removed. For independent positioning of the FluidFM probe with respect to the cell dish, the AFM scan head (Nanosurf AG, Liestal, Switzerland) was elevated above the standard microscopy stage using a custom mount attached to the microscope’s incubator box. This mount, with an integrated manual *x*–*y*-positioning system, hovered approximately 0.5 mm above the microscopy stage. To reach the substrate surface despite the elevated AFM scan head, adjustments were made to the z-screw holders and z-motor housing, and magnets were placed to lower the FluidFM probe by around 5 mm. The cell dish was positioned on a spaced sample-holder, allowing access for microscopy using a 63x oil objective with a numerical aperture of 1.4 (HC PL APO CS2, Leica Microsystems GmbH, Wetzlar, Germany). AFM laser alignment was monitored using an ocular camera (DinoEye, AnMo Electronics Co, Taipei, Taiwan). FLIM imaging utilized laser (488 nm) pulses operating at a frequency of 40 MHz. The microscope chamber was maintained at a temperature of 37°C. The AFM piezo movement, deflection of the cantilever, and fluidic pressure (in the case of injection in mechano-chemical stimulation) were tracked by recording timelines using a data acquisition box and a custom LabView script (National Instruments Co, Austin, USA). These timelines were then correlated with the recorded lifetime images through a trigger output from the confocal microscope.

### Mechanical stimulation

For the mechanical stimulation, FluidFM probes were approached on the ER Flipper-TR-stained HeLa cells. The set point for the indentation force varied between 2 and 100 nN, while the approach speed was set at 1 µm s^–1^. Simultaneously, lifetime images of the target cell were captured with a field of view measuring 92.35 µm × 23.09 µm, featuring a pixel size of 90 nm. These images were scanned at a speed of 100 Hz, utilizing a pinhole aperture of 1.0 airy unit (equivalent to 95.5 µm). Photons from 30 frames per image were collected and averaged, with an acquisition time of 40 s for each image. For comparison, one image was taken before the mechanical stimuli, one during the pause time of the stimulus, and one after removing the probe.

### Mechano-chemical stimulation

For mechano-chemical stimulation, FluidFM probes filled with the desired chemical solution were approached on the ER Flipper-TR-stained HeLa cells with varied levels of lamin proteins. The setpoint was adjusted to 150 nN for the indentation to ensure a complete insertion of the probe aperture inside the cell, thereby a sealed injection, with an approach speed of 1 µm s^–1^. After reaching the setpoint and undergoing a brief waiting period, an injection pulse of 100 mbar for 10 s was administered. The injection parameters, comprising the indentation force, approach speed, and fluidic pressure pulse, had been specifically calibrated for optimal application to HeLa cells beforehand. Concurrently, lifetime images of the target cell were acquired for up to 5 min post-injection, covering a field of view spanning 46.13 µm x 5.69 µm and featuring a pixel size of 90 nm. These images underwent scanning at a rate of 200 Hz, utilizing a pinhole aperture of 1.0 airy unit (equivalent to 95.5 µm). Each image was composed of photons collected from 100 frames, which were then averaged, with an acquisition time of 18 s for each image. For comparison, lifetime images were captured before the stimulus, during the pause time of the mechanical stimulus before injection pulse (‘mech’), series of images after injection pulse (‘mech + inj’), and one after removing the probe. The chosen observation period of up to 5 min post-injection was defined by the expected rapid pharmacokinetics of the drugs upon direct intracellular delivery. To achieve this, a high-concentration stock (50 µM) of CytoD and Nocodazole was used to yield a final intracellular concentration of 0.5 µM. This physiologically relevant dose was anticipated to act as fast as within 3 min for CytoD and within 5 min for Nocodazole, a timing crucial for our study’s focus on the early dynamic response and initial deviation from control conditions rather than the steady state achieved at longer time points. This time estimation is firmly based on reported values in the literature. For example, a recent comprehensive study quantified actin dynamics upon CytoD application using TIRF microscopy and reported the onset of actin polymerization inhibition to occur approximately 150 s after introducing 5 nM CytoD (Mitani et al., 2025). Similarly, another study demonstrated the rapid disassembly of MTs in monocytes incubated with 1 µM Nocodazole, reporting a half-time of 40 s for complete disassembly (Cassimeris et al., 1986).

For experiments studying the relaxation behavior, an additional lifetime image was captured 5 min post-stimulus.

### FLIM data analysis

Firstly, different ROIs of the ER, nuclear membrane (Nuc-mem), and the indentation site (Indent-site) (a region around the indenting probe) were manually selected (Figure 1—figure supplement 1). The circular structure enveloping the nucleus as detected in lifetime images of cells stained with ER Flipper-TR (Figure 1C) was attributed to the ROI associated with the nuclear membrane. To validate this correlation with the nuclear membrane, cells were co-stained with NucBlue (DNA-binding dye) and ER Flipper-TR. The resulting images captured the overlapping signals (Figure 1—figure supplement 2). The substantial overlap observed affirms the accuracy of the defined ROI as the nuclear membrane in this study. A two-exponential reconvolution fit was conducted on the time-correlated single-photon counting histogram obtained for every pixel within selected ROIs in all lifetime images. This process allowed for the determination of the two components of the ER Flipper-TR lifetime, with the smaller component being fixed for each image and disregarded for all subsequent analyses. The larger component, which is directly related to membrane tension, was determined for each pixel, resulting in the spatial distribution of membrane tension during mechanical or mechano-chemical stimulation. Previously, we verified that changes in the larger component of Flipper-TR lifetime during FluidFM stimulation were mainly associated with alterations in tension rather than lipid order (Lüchtefeld et al., 2024). For that, we employed the Laurdan-staining assay, which utilizes Laurdan, a fluorescent dye sensitive to variations in lipid organization within the membrane. By applying a threshold to the fluorescence intensity of each pixel, membranal structures were distinguished from the background. Subsequently, the amplitude-based average lifetime $\tau _{amp}$ was calculated (Equation 1) for a selected region of interest encompassing the ER, nuclear membrane, and indentation site,

(1) $$\displaystyle  \tau _{amp}=\frac{\sum _{i}A_{i}\times \tau _{i}}{\sum _{i}A_{i}}$$

where $A_{i}$ represents the amplitude and $\tau _{i}$ denotes the lifetime for the *i*th pixel.

The mean lifetime values were determined for the periods before, during, and after the stimulus across all ROIs. Subsequently, the value associated with the pre-stimulus state of each cell was subtracted from its respective image for the during and after periods, resulting in the calculation of lifetime change values. The obtained fluorescence lifetimes are associated with membrane tension rather than lipid composition, as examined in our previous study (Lüchtefeld et al., 2024). However, the conversion factor for ER Flipper-TR was neither addressed previously nor studied here due to the challenges in establishing an experimental setup to measure the ER tension in intact cells. Therefore, the lifetime values are reported directly.

### Statistical analysis

Data were tested for normal distribution using the Shapiro–Wilk test before applying parametric statistical methods. Differences between two groups were assessed using an unpaired two-tailed Student’s *t*-test. Where multiple comparisons were conducted, a *t*-test with Holm correction was applied to control the false discovery rates, as indicated in the relevant sections. p-values were calculated and reported for all analyses, with a significance threshold set at p < 0.05. The sample size for each experiment (*n*) is provided in the figure legends, and the number of independent biological replicates (*N*) is explicitly reported to ensure reproducibility. *N* represents the number of independent biological replicates, while *n* represents the number of individual cells analyzed. Data points were excluded when stable FluidFM probe-cell contact at the specified force could not be maintained due to technical failure or cell escape during the experiment. Exclusion was confirmed based on force measurement traces and/or loss of imaging focus during FLIM acquisition. All statistical analyses were performed using custom Python scripts.

## Data Availability

The datasets generated and analyzed during the current study, including the FLIM image data and the Python scripts developed for the quantification of values, statistical analysis, and data visualization, are available in the ETH Zürich Research Collection at https://doi.org/10.3929/ethz-c-000790250. The following dataset was generated: Zare-EelanjeghE LewisRTM LüchtefeldI KutayU ZambelliT 2026Quantifying Intracellular Mechanosensitive Response upon Spatially Defined Mechano-Chemical TriggeringETH Library research collection10.3929/ethz-c-000790250PMC1327506342307999

## Additional files

MDAR checklist
