# Supplementary material for: Quantifying intracellular mechanosensitive response upon spatially defined mechano-chemical triggering
Source: eLife. 2026 Jun 17;14:RP107220. doi: 10.7554/eLife.107220 (PMC13275063; doi:10.7554/eLife.107220)
Supplement: Figure 4—figure supplement 1—source data 1. [file elife-107220-fig4-figsupp1-data1.zip › Figure_4-figure_supplement_1-source_data_1.pdf]

Figure 4-figure supplement 1-source data 1

Samples were blotted on three separate gels.

Replicate 1

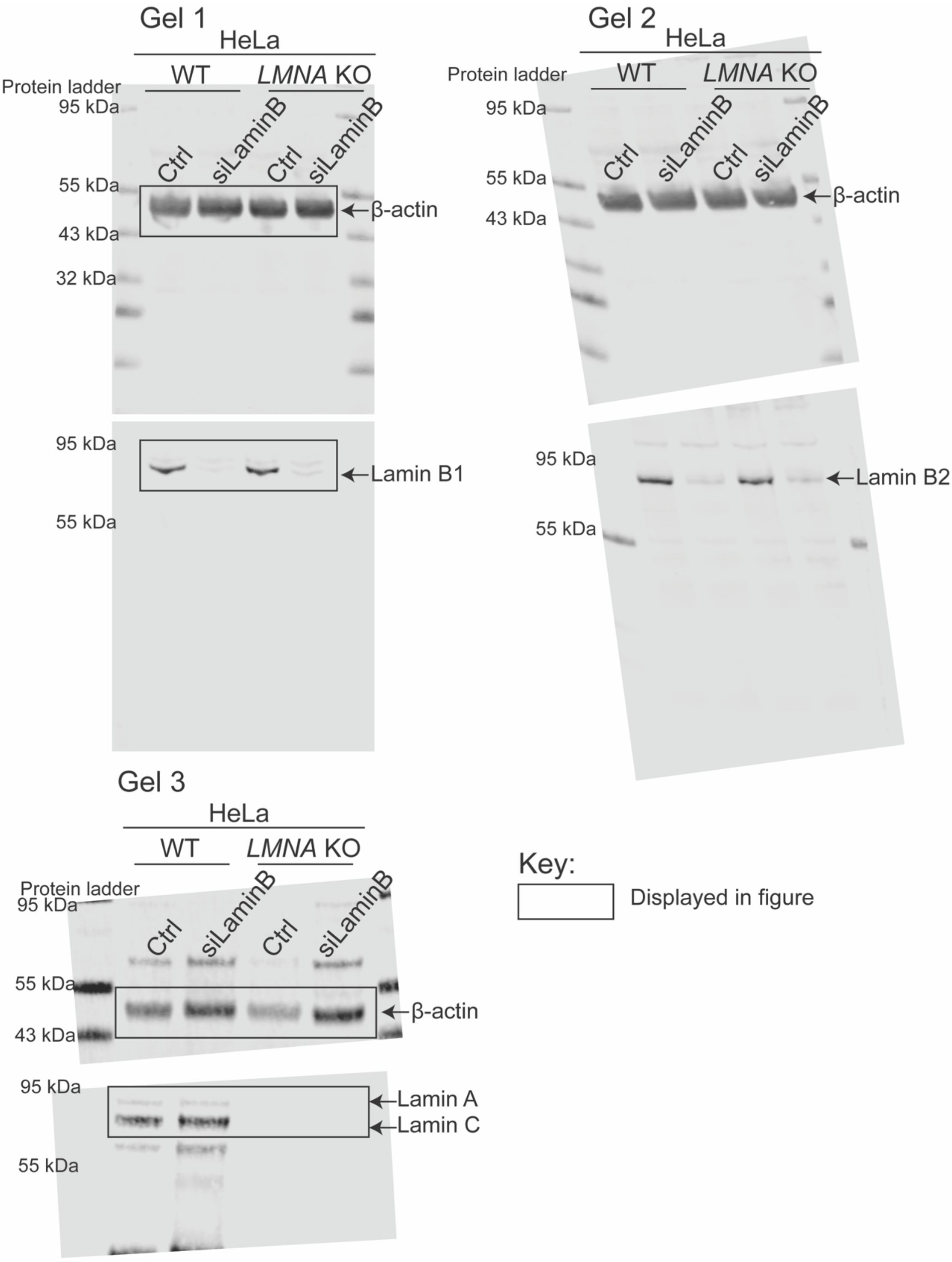

Replicate 2

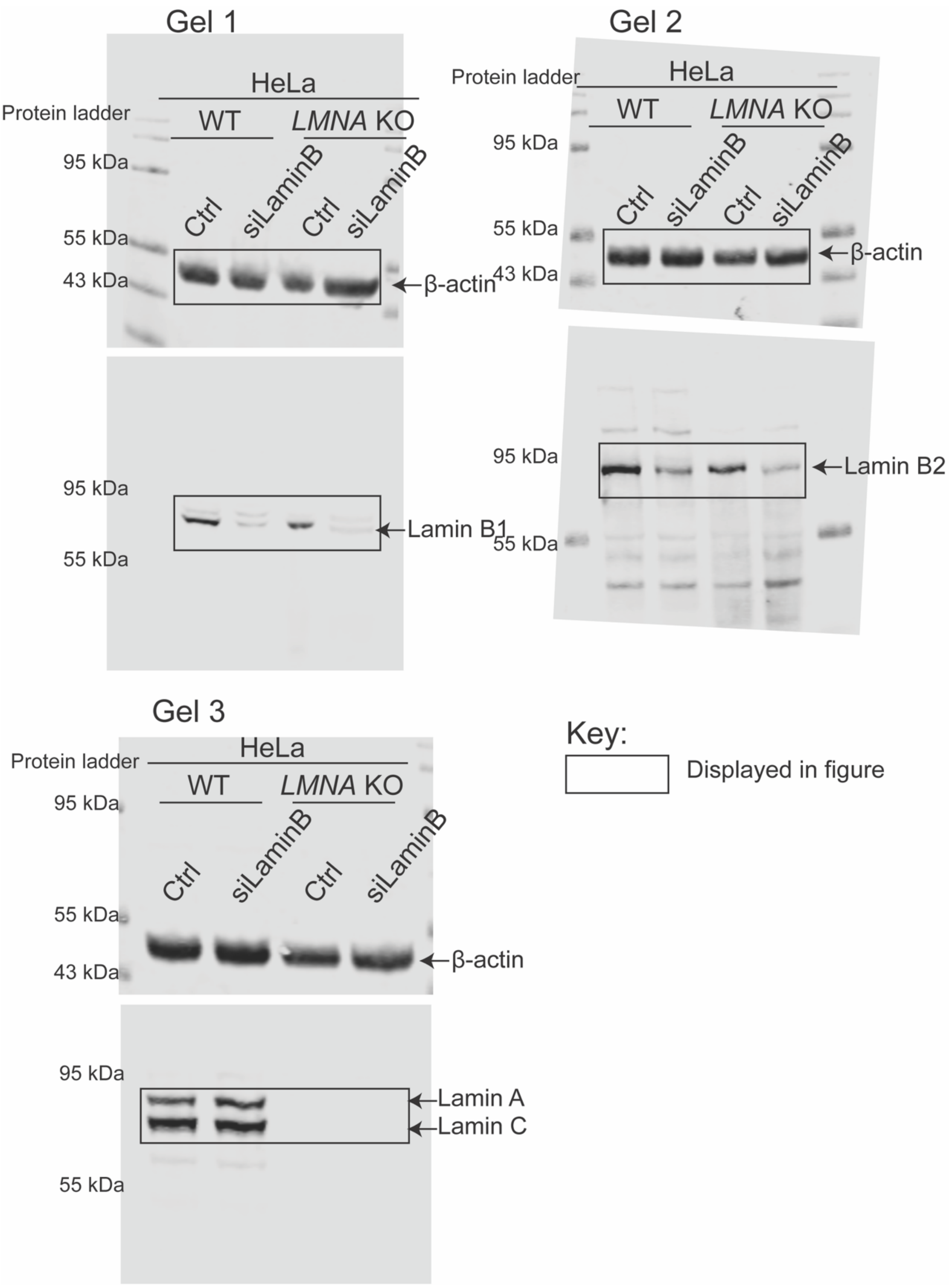

Gel 1
Protein ladder
HeLa
WT
LMNA KO
Ctrl
siLaminB
Ctrl
siLaminB
95 kDa
55 kDa
43 kDa
β-actin
95 kDa
55 kDa
Lamin B1
Gel 2
Protein ladder
HeLa
WT
LMNA KO
Ctrl
siLaminB
Ctrl
siLaminB
95 kDa
55 kDa
43 kDa
β-actin
95 kDa
55 kDa
Lamin B2
Gel 3
Protein ladder
HeLa
WT
LMNA KO
Ctrl
siLaminB
Ctrl
siLaminB
95 kDa
55 kDa
43 kDa
β-actin
95 kDa
55 kDa
Lamin A
Lamin C
Key:
Displayed in figure

Replicate 3

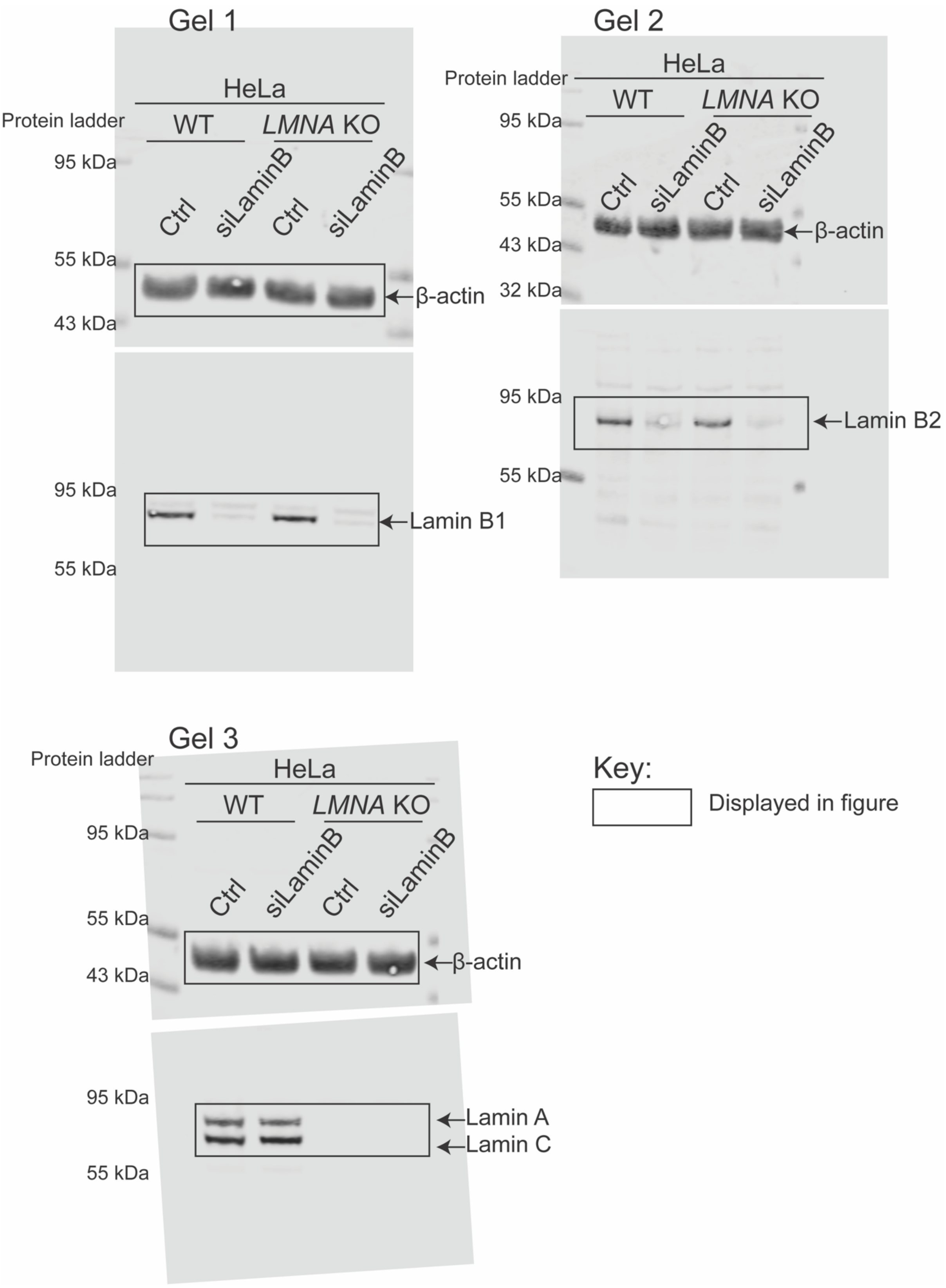

Gel 1
Protein ladder
HeLa
WT
LMNA KO
95 kDa
Ctrl
siLaminB
Ctrl
siLaminB
55 kDa
β-actin
43 kDa
95 kDa
Lamin B1
55 kDa
Gel 2
Protein ladder
HeLa
WT
LMNA KO
95 kDa
Ctrl
siLaminB
Ctrl
siLaminB
55 kDa
β-actin
43 kDa
32 kDa
95 kDa
Lamin B2
55 kDa
Gel 3
Protein ladder
HeLa
WT
LMNA KO
95 kDa
Ctrl
siLaminB
Ctrl
siLaminB
55 kDa
β-actin
43 kDa
95 kDa
Lamin A
Lamin C
55 kDa
Key:
Displayed in figure
