# Supplementary figures and images for: Quantifying intracellular mechanosensitive response upon spatially defined mechano-chemical triggering
Source: eLife. 2026 Jun 17;14:RP107220. doi: 10.7554/eLife.107220 (PMC13275063; doi:10.7554/eLife.107220)

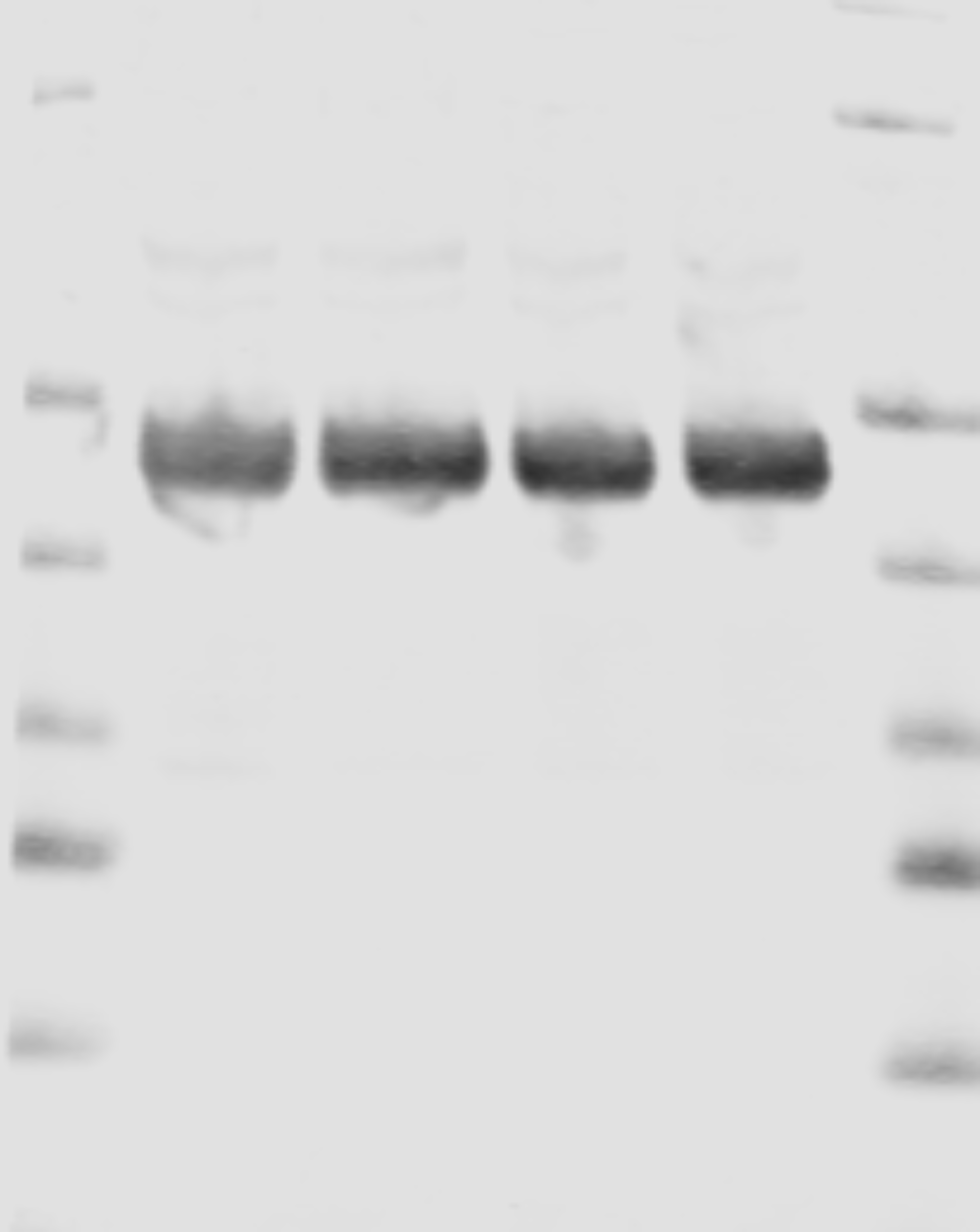

Supplement: Figure 4—figure supplement 1—source data 2. [file elife-107220-fig4-figsupp1-data2.zip › Figure 4-figure supplement 1-source data 2/Figure 4-figure supplement 1-source data 2/Rep 1/BetaActin_gel 1tif.tif]

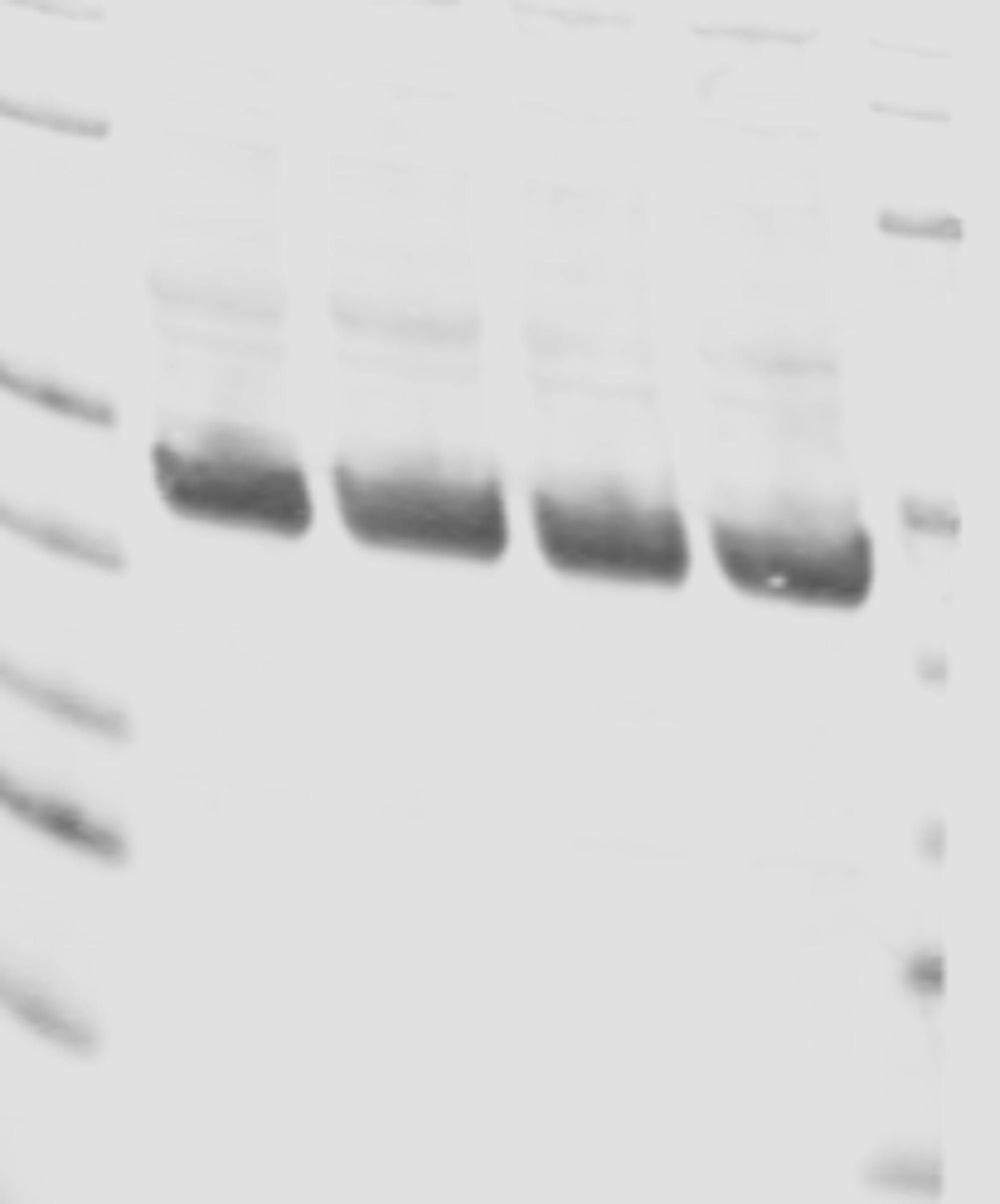

Supplement: Figure 4—figure supplement 1—source data 2. [file elife-107220-fig4-figsupp1-data2.zip › Figure 4-figure supplement 1-source data 2/Figure 4-figure supplement 1-source data 2/Rep 1/BetaActin_gel 2.tif]

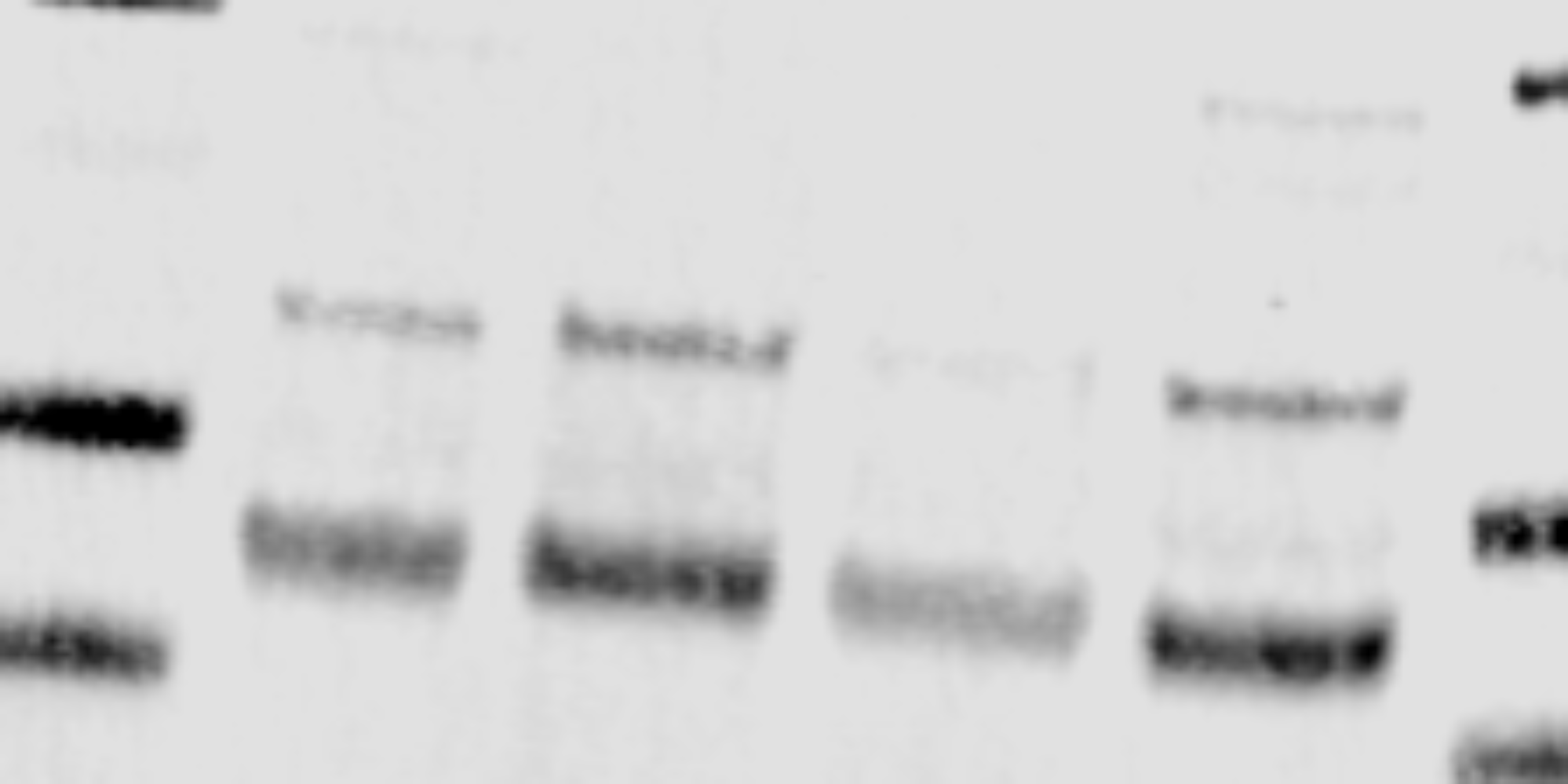

Supplement: Figure 4—figure supplement 1—source data 2. [file elife-107220-fig4-figsupp1-data2.zip › Figure 4-figure supplement 1-source data 2/Figure 4-figure supplement 1-source data 2/Rep 1/BetaActin_gel 3.tif]

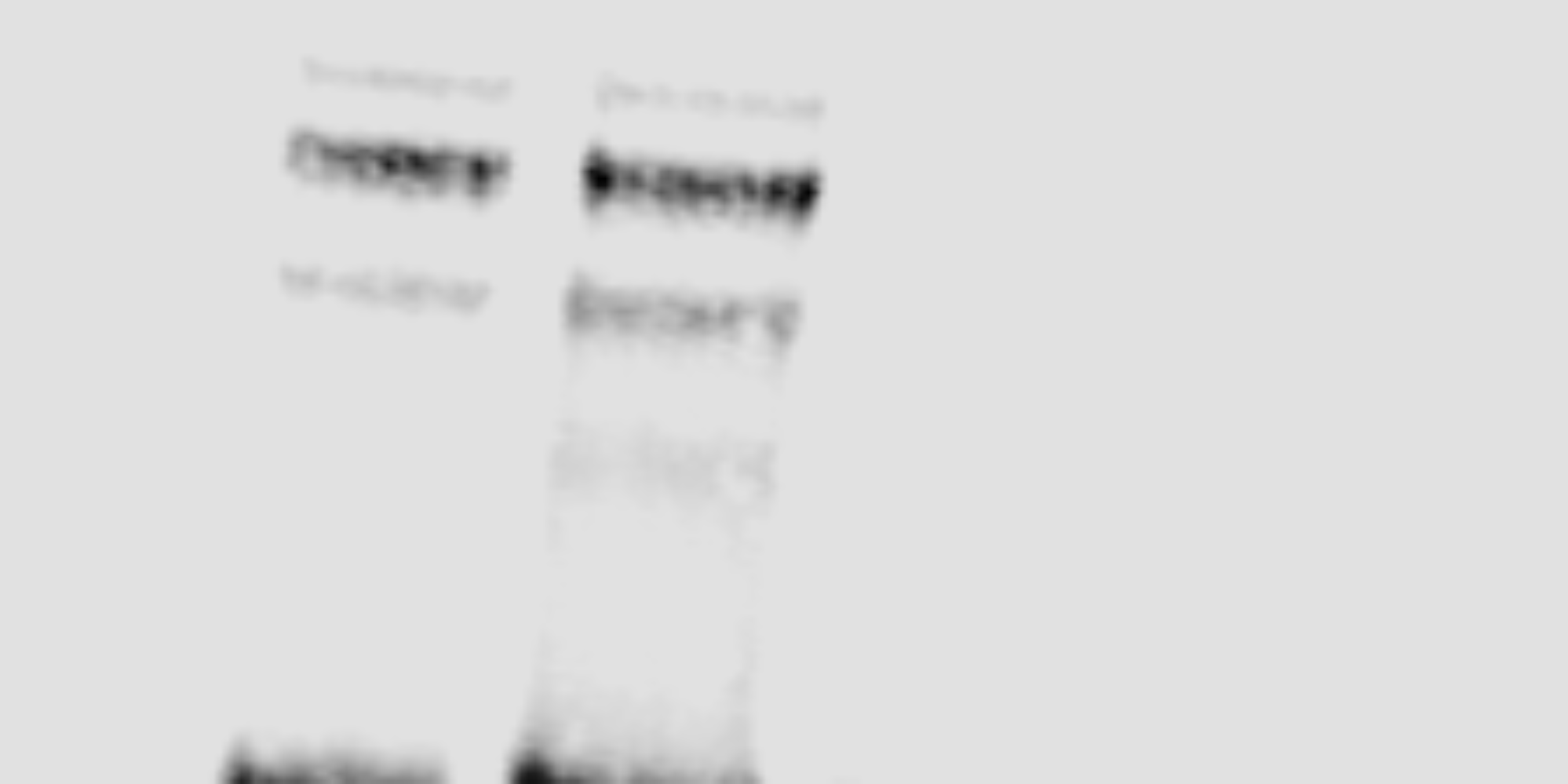

Supplement: Figure 4—figure supplement 1—source data 2. [file elife-107220-fig4-figsupp1-data2.zip › Figure 4-figure supplement 1-source data 2/Figure 4-figure supplement 1-source data 2/Rep 1/lamin A_C.tif]

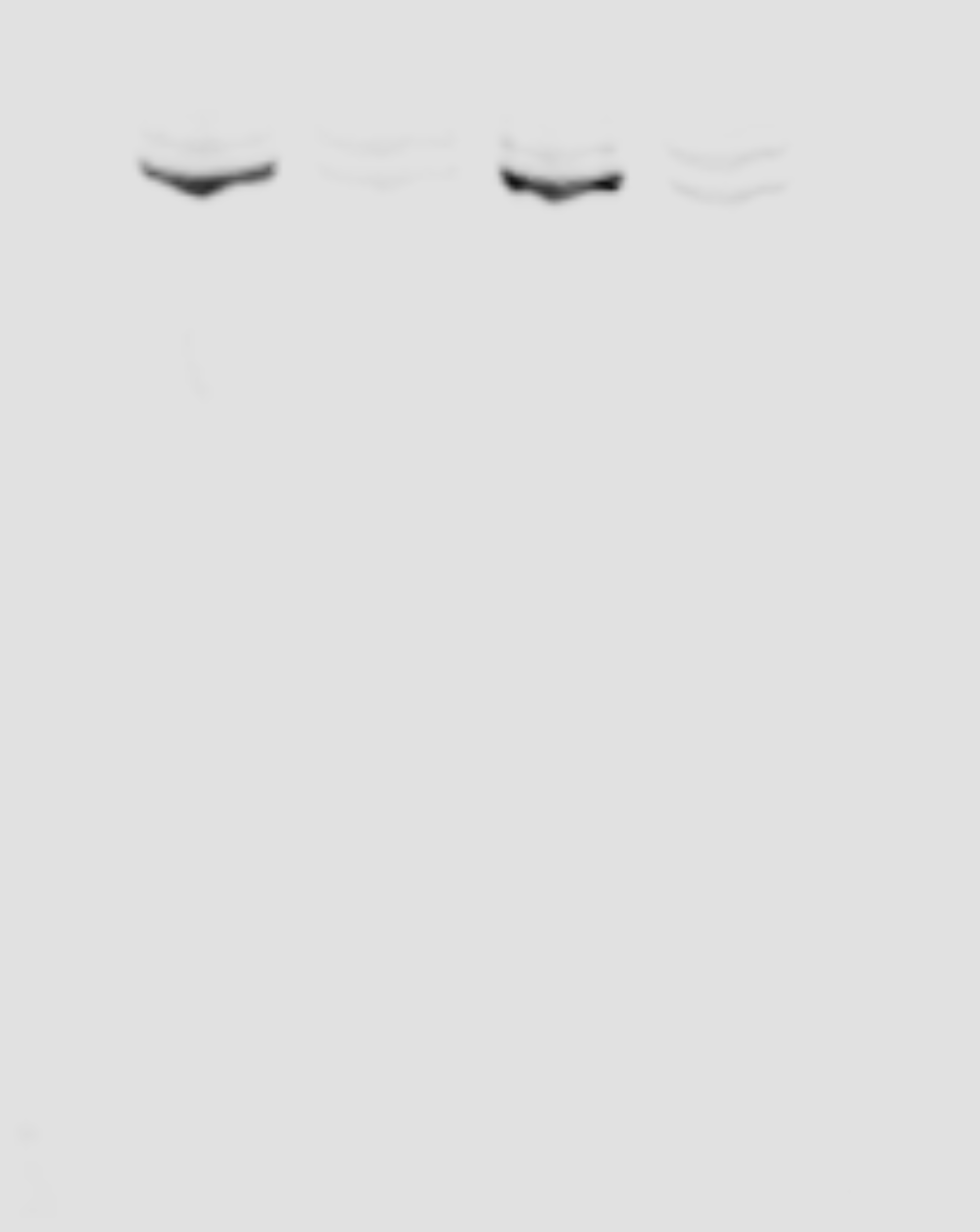

Supplement: Figure 4—figure supplement 1—source data 2. [file elife-107220-fig4-figsupp1-data2.zip › Figure 4-figure supplement 1-source data 2/Figure 4-figure supplement 1-source data 2/Rep 1/lamin B1.tif]

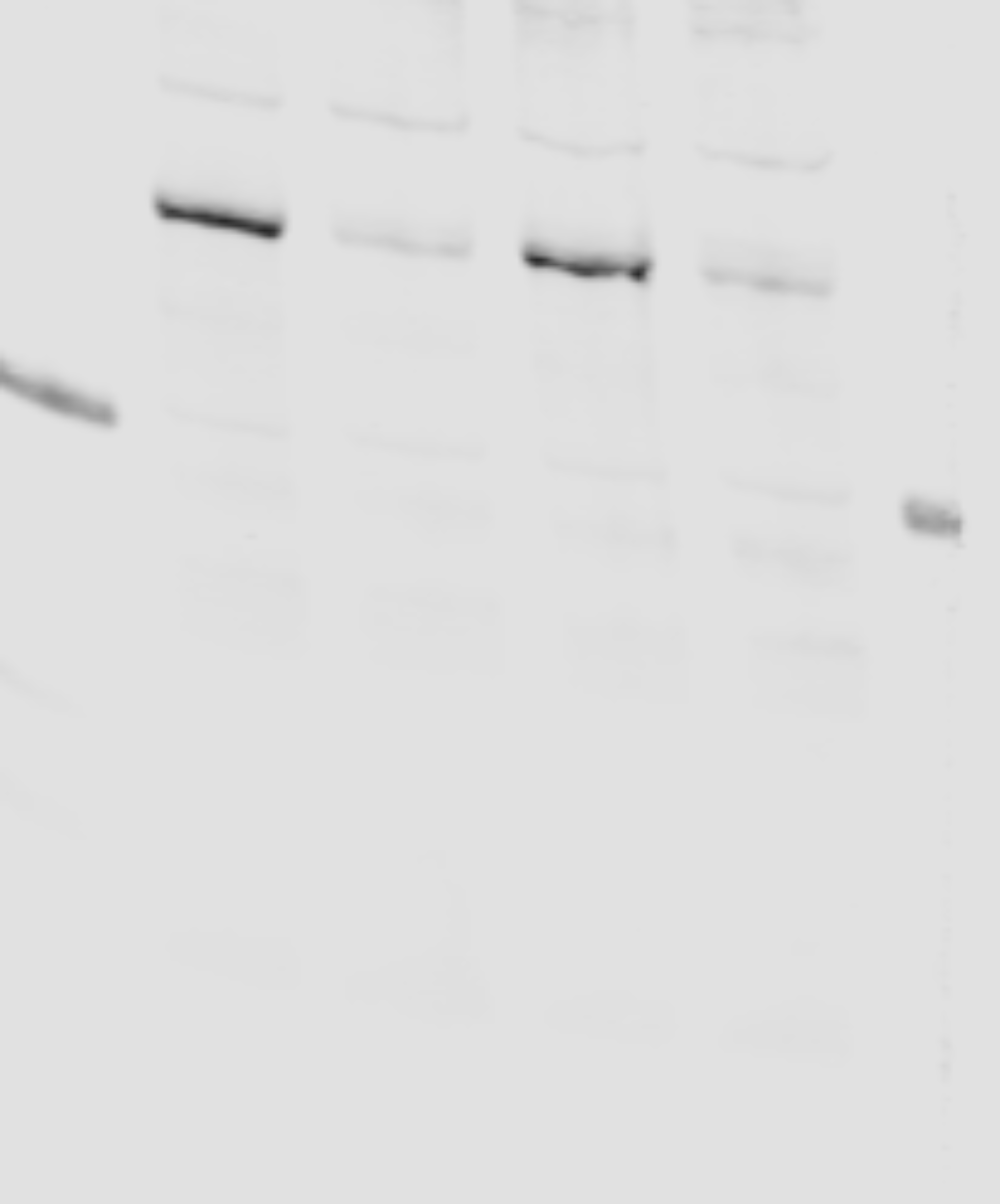

Supplement: Figure 4—figure supplement 1—source data 2. [file elife-107220-fig4-figsupp1-data2.zip › Figure 4-figure supplement 1-source data 2/Figure 4-figure supplement 1-source data 2/Rep 1/lamin B2.tif]

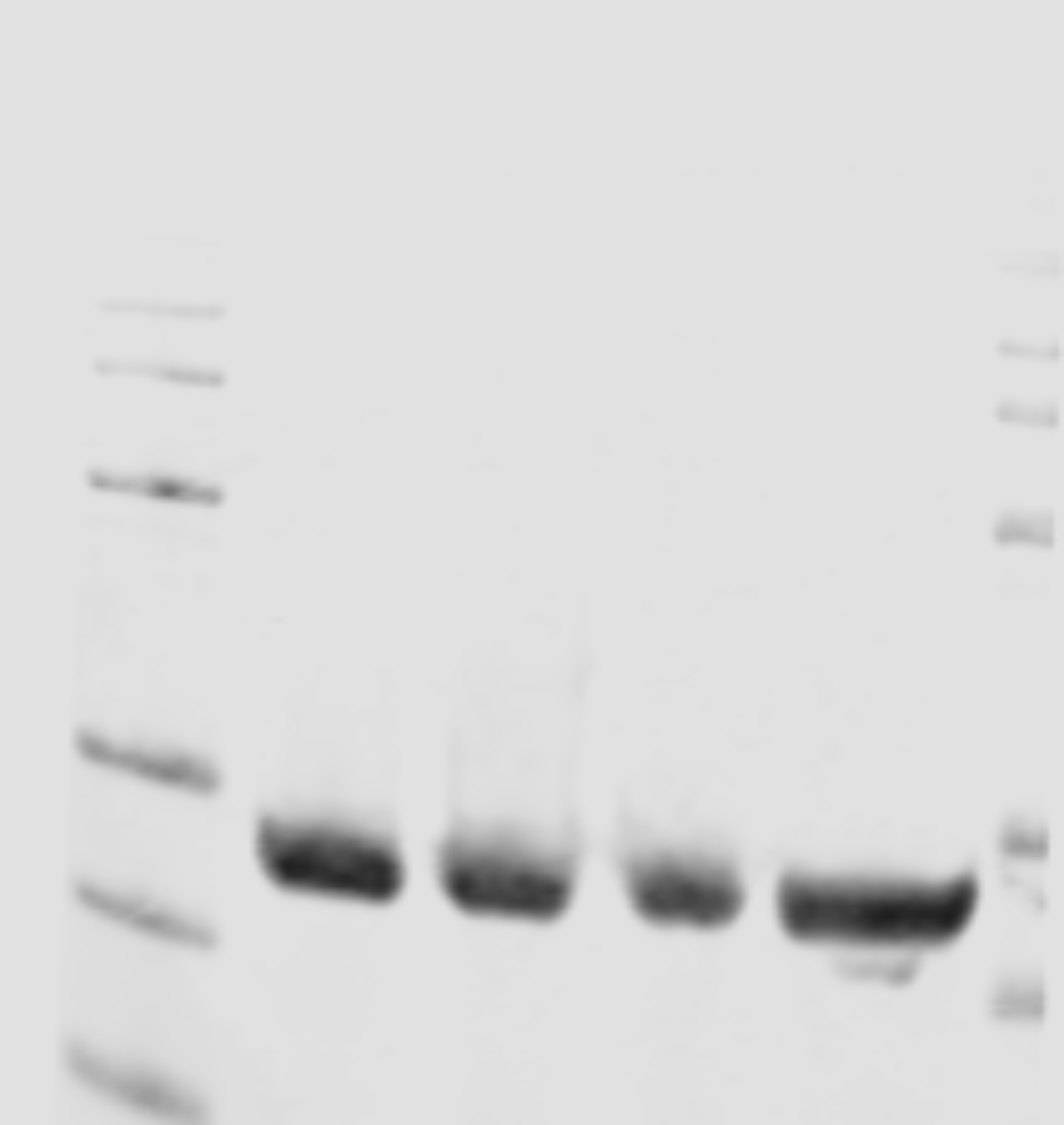

Supplement: Figure 4—figure supplement 1—source data 2. [file elife-107220-fig4-figsupp1-data2.zip › Figure 4-figure supplement 1-source data 2/Figure 4-figure supplement 1-source data 2/Rep 2/BetaActin_Gel 1.tif]

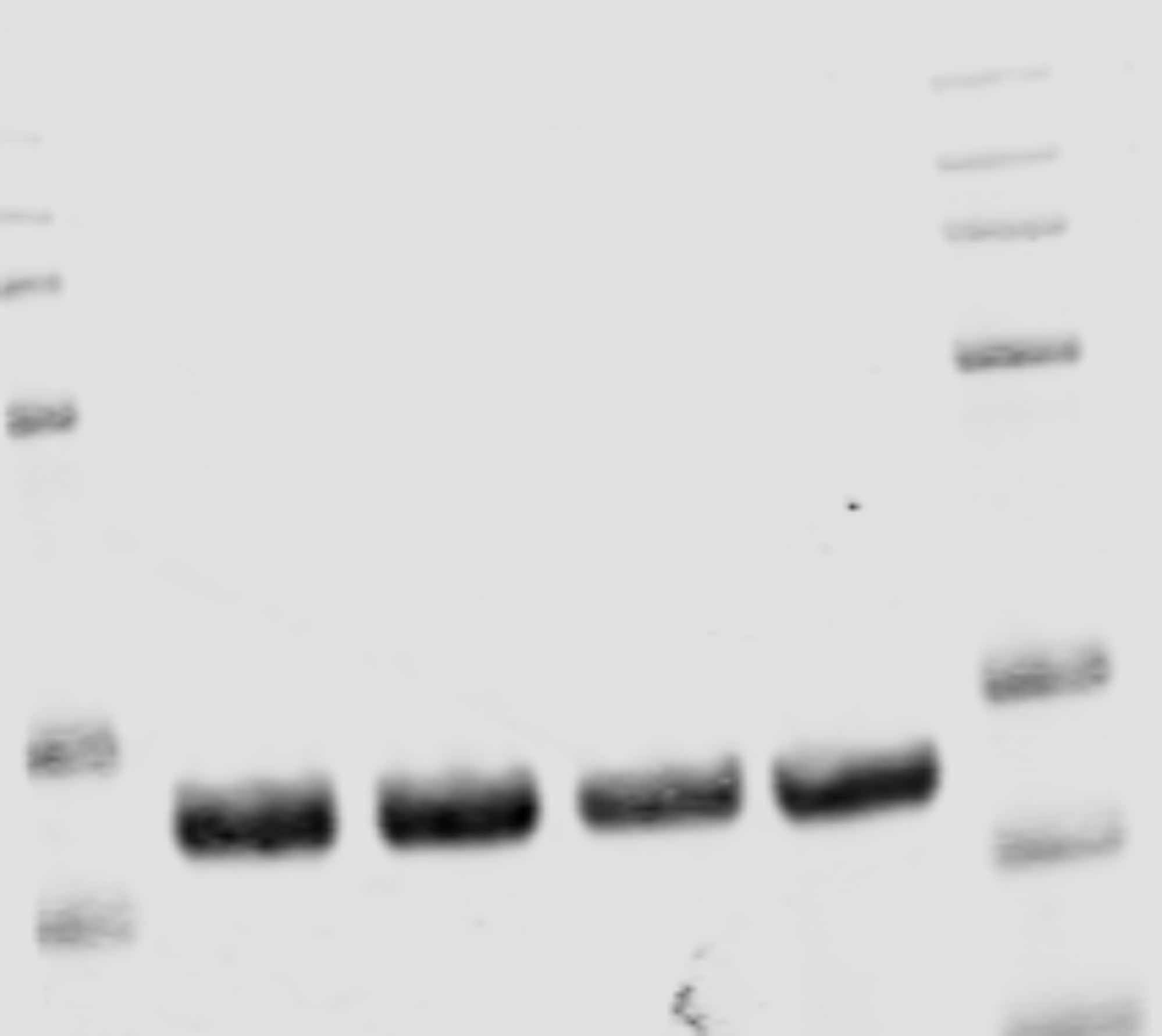

Supplement: Figure 4—figure supplement 1—source data 2. [file elife-107220-fig4-figsupp1-data2.zip › Figure 4-figure supplement 1-source data 2/Figure 4-figure supplement 1-source data 2/Rep 2/BetaActin_Gel 2.tif]

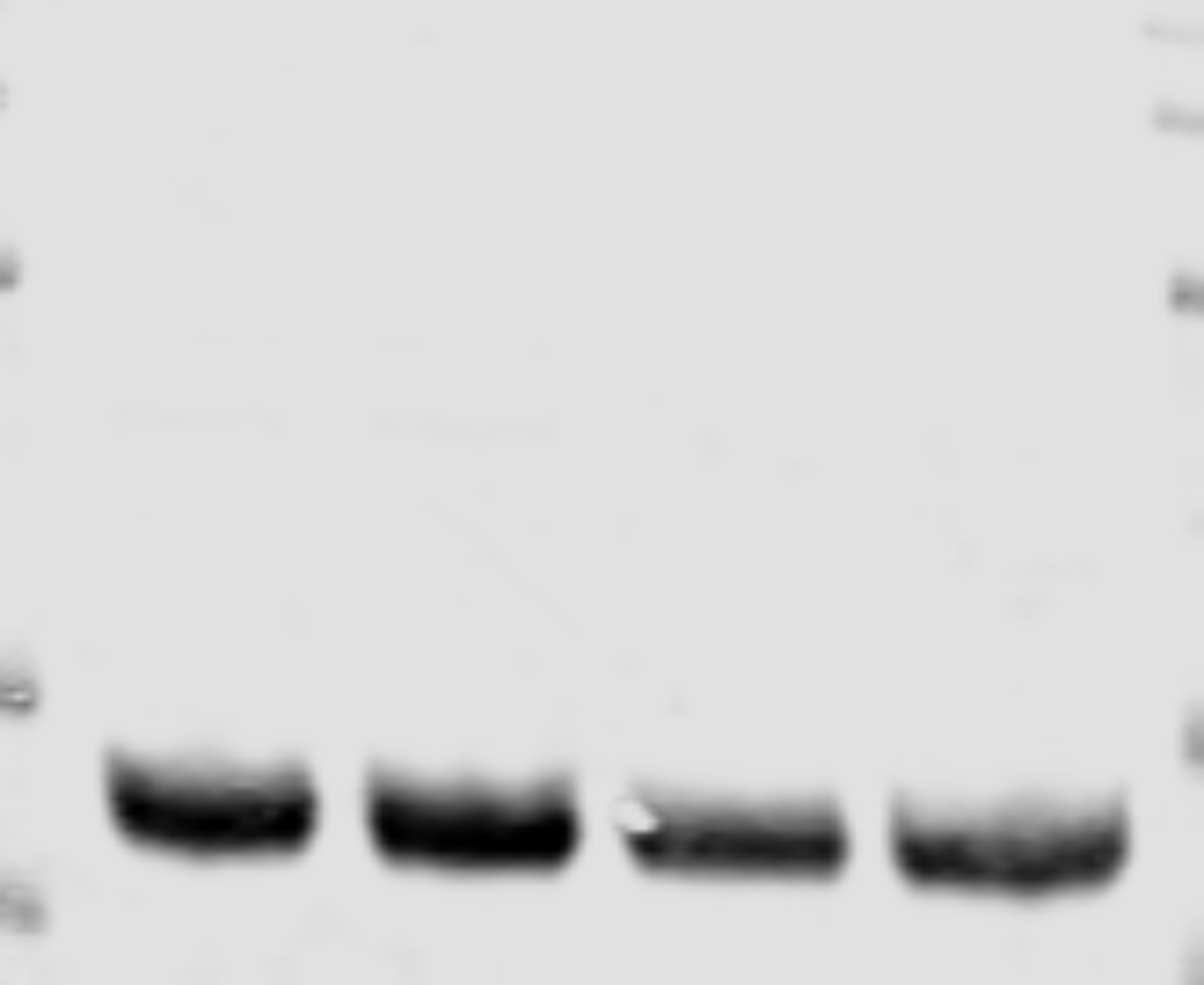

Supplement: Figure 4—figure supplement 1—source data 2. [file elife-107220-fig4-figsupp1-data2.zip › Figure 4-figure supplement 1-source data 2/Figure 4-figure supplement 1-source data 2/Rep 2/BetaActin_Gel 3.tif]

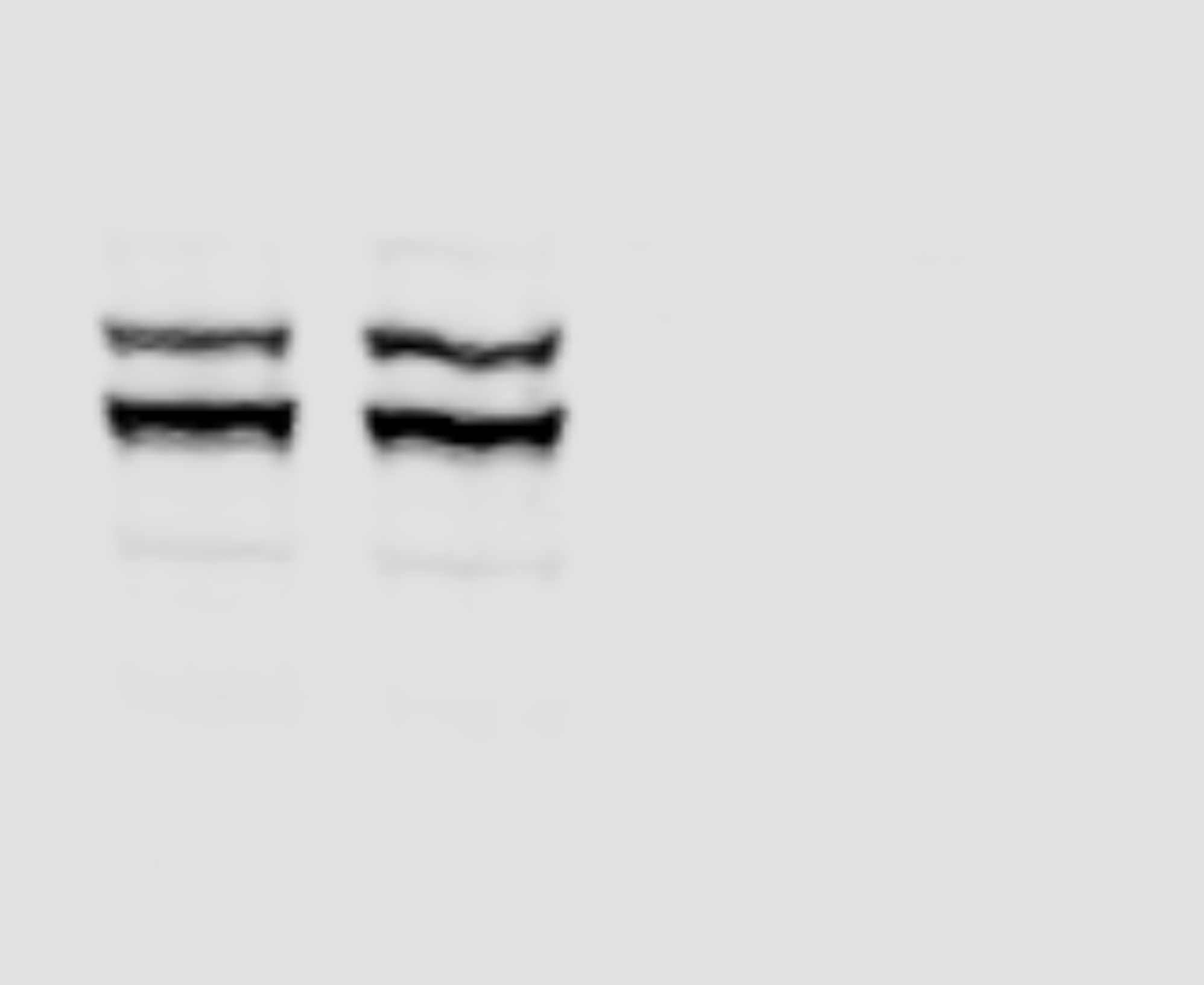

Supplement: Figure 4—figure supplement 1—source data 2. [file elife-107220-fig4-figsupp1-data2.zip › Figure 4-figure supplement 1-source data 2/Figure 4-figure supplement 1-source data 2/Rep 2/Lamin A_C.tif]

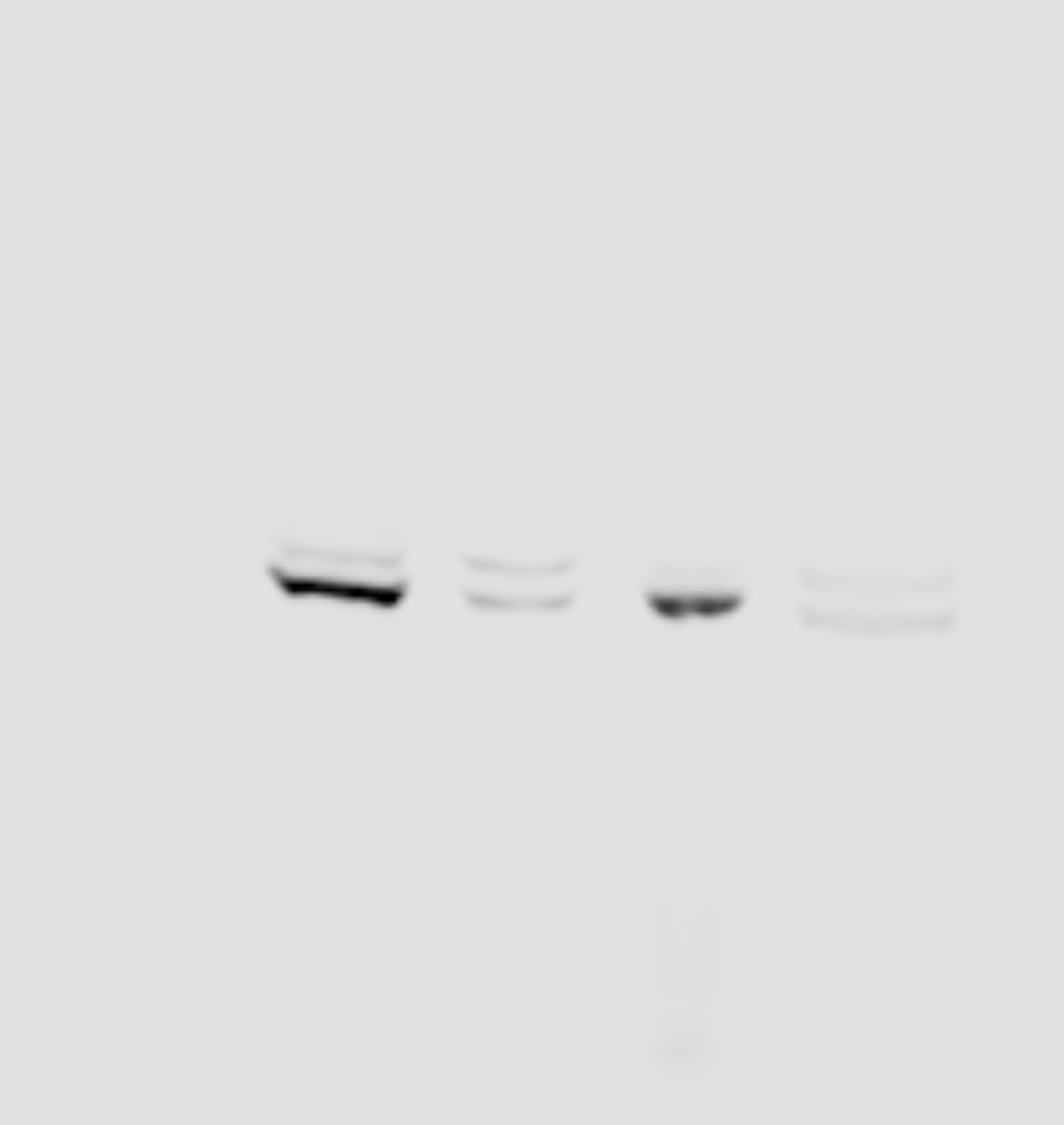

Supplement: Figure 4—figure supplement 1—source data 2. [file elife-107220-fig4-figsupp1-data2.zip › Figure 4-figure supplement 1-source data 2/Figure 4-figure supplement 1-source data 2/Rep 2/Lamin B1.tif]

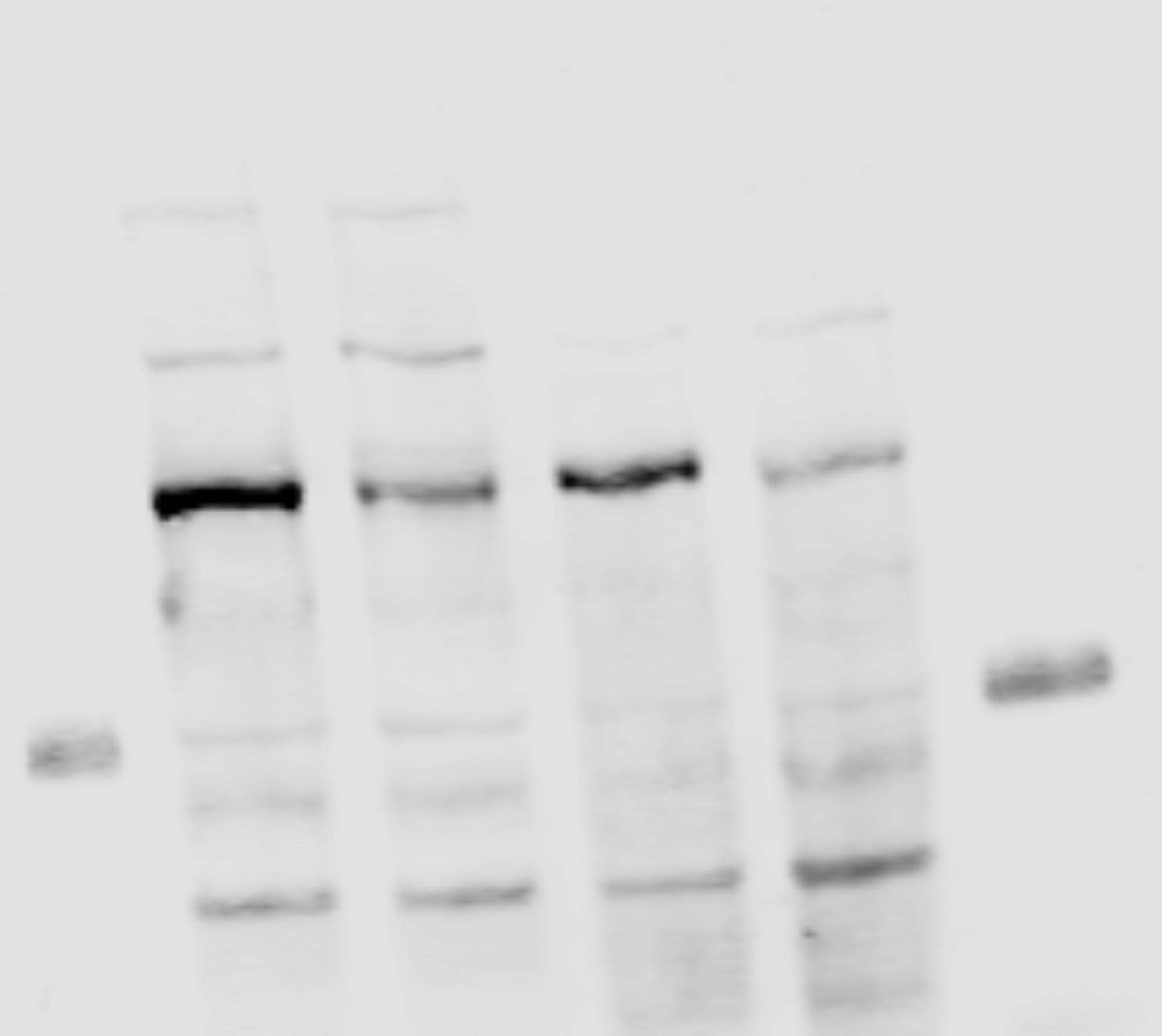

Supplement: Figure 4—figure supplement 1—source data 2. [file elife-107220-fig4-figsupp1-data2.zip › Figure 4-figure supplement 1-source data 2/Figure 4-figure supplement 1-source data 2/Rep 2/Lamin B2.tif]

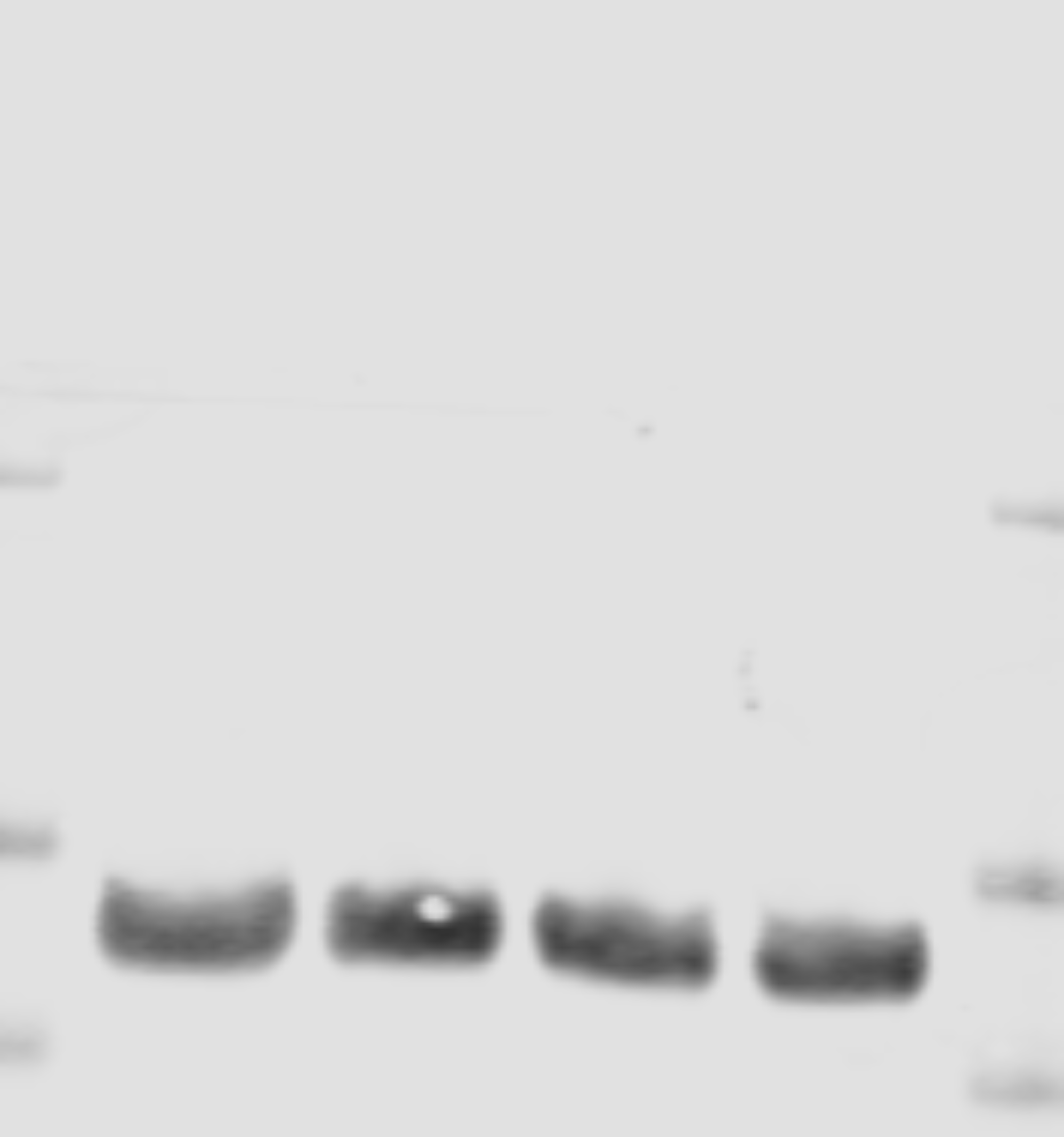

Supplement: Figure 4—figure supplement 1—source data 2. [file elife-107220-fig4-figsupp1-data2.zip › Figure 4-figure supplement 1-source data 2/Figure 4-figure supplement 1-source data 2/Rep 3/BetaActin_Gel 1.tif]

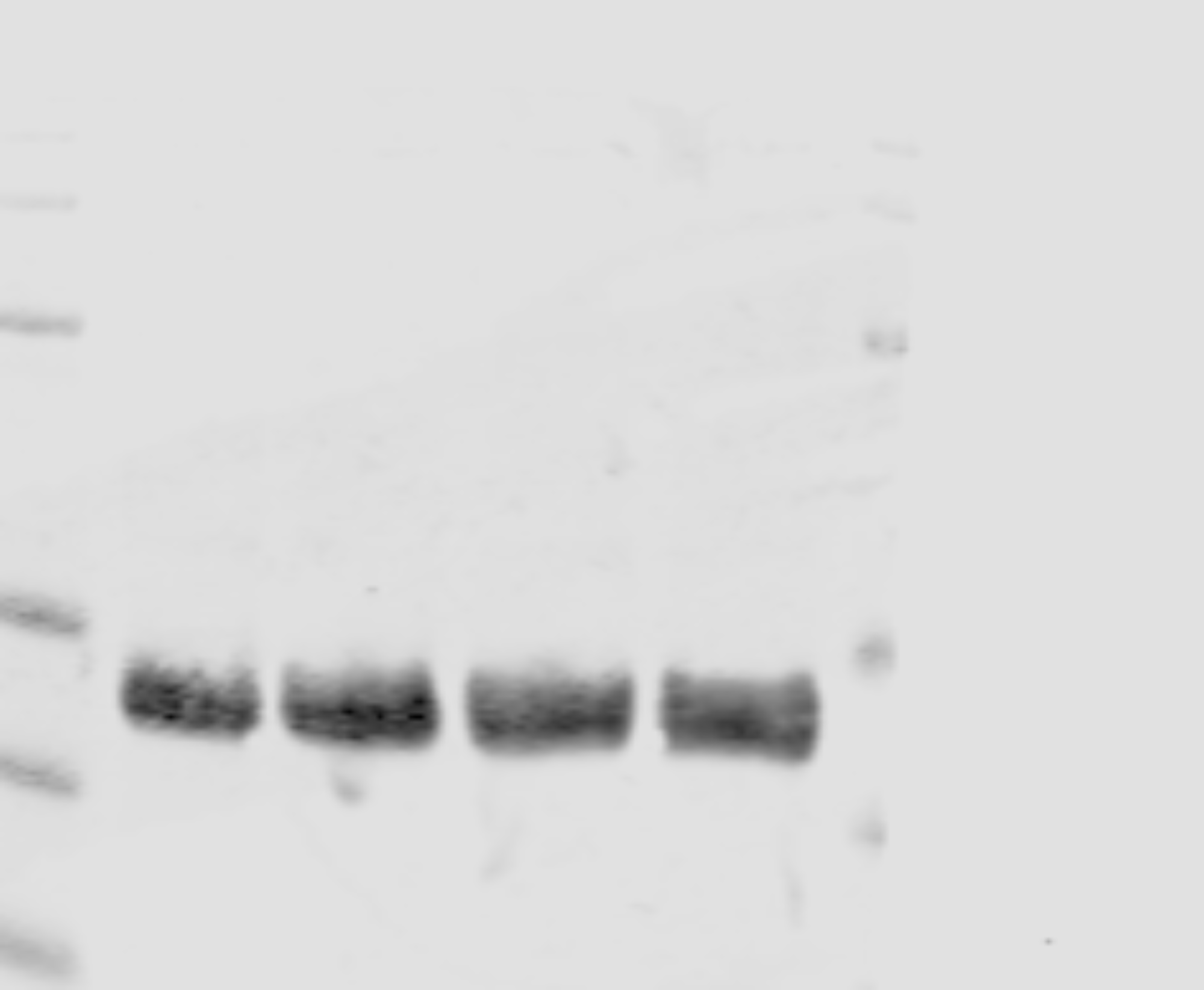

Supplement: Figure 4—figure supplement 1—source data 2. [file elife-107220-fig4-figsupp1-data2.zip › Figure 4-figure supplement 1-source data 2/Figure 4-figure supplement 1-source data 2/Rep 3/BetaActin_Gel 2.tif]

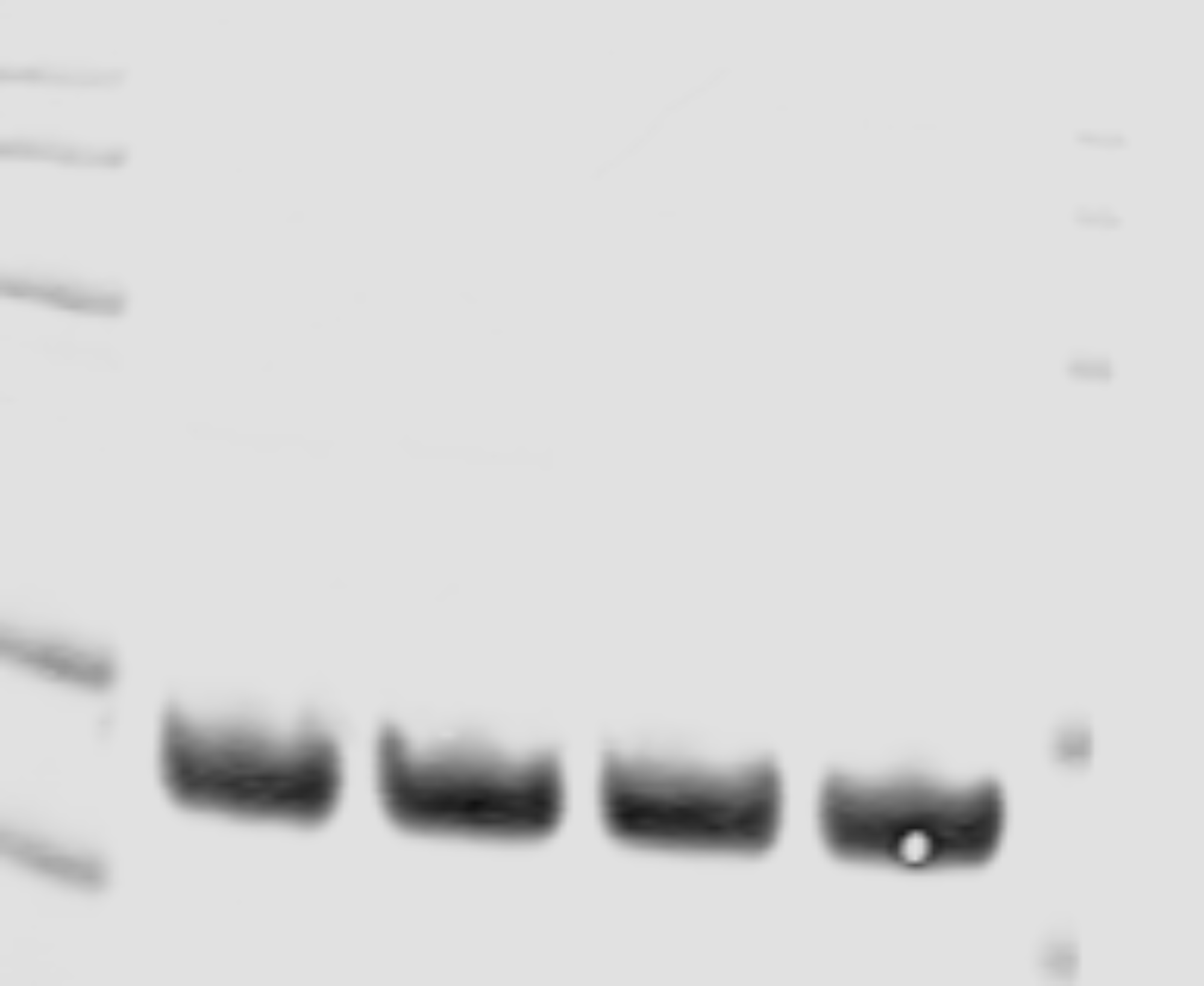

Supplement: Figure 4—figure supplement 1—source data 2. [file elife-107220-fig4-figsupp1-data2.zip › Figure 4-figure supplement 1-source data 2/Figure 4-figure supplement 1-source data 2/Rep 3/BetaActin_Gel 3.tif]

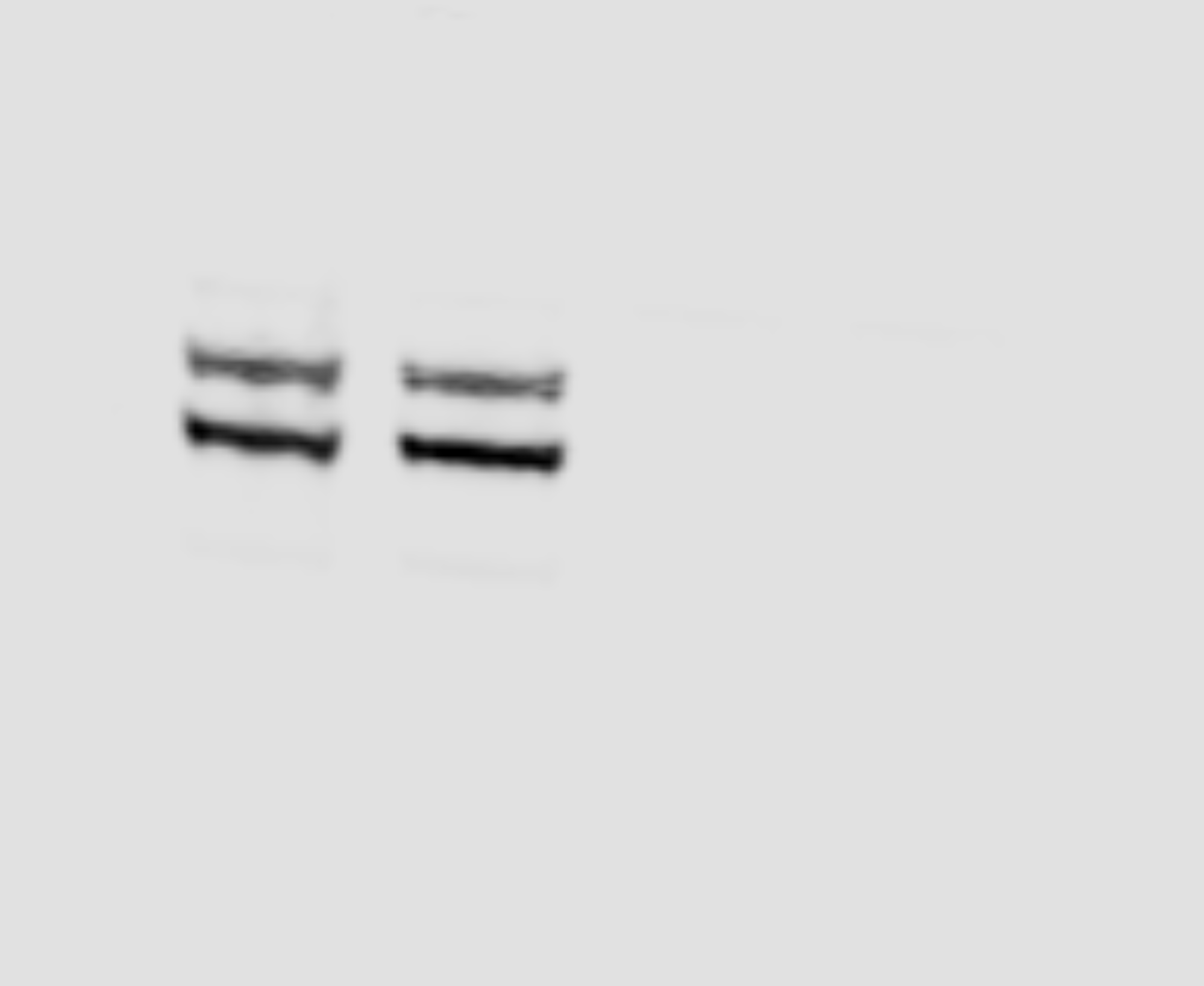

Supplement: Figure 4—figure supplement 1—source data 2. [file elife-107220-fig4-figsupp1-data2.zip › Figure 4-figure supplement 1-source data 2/Figure 4-figure supplement 1-source data 2/Rep 3/Lamin A_C.tif]

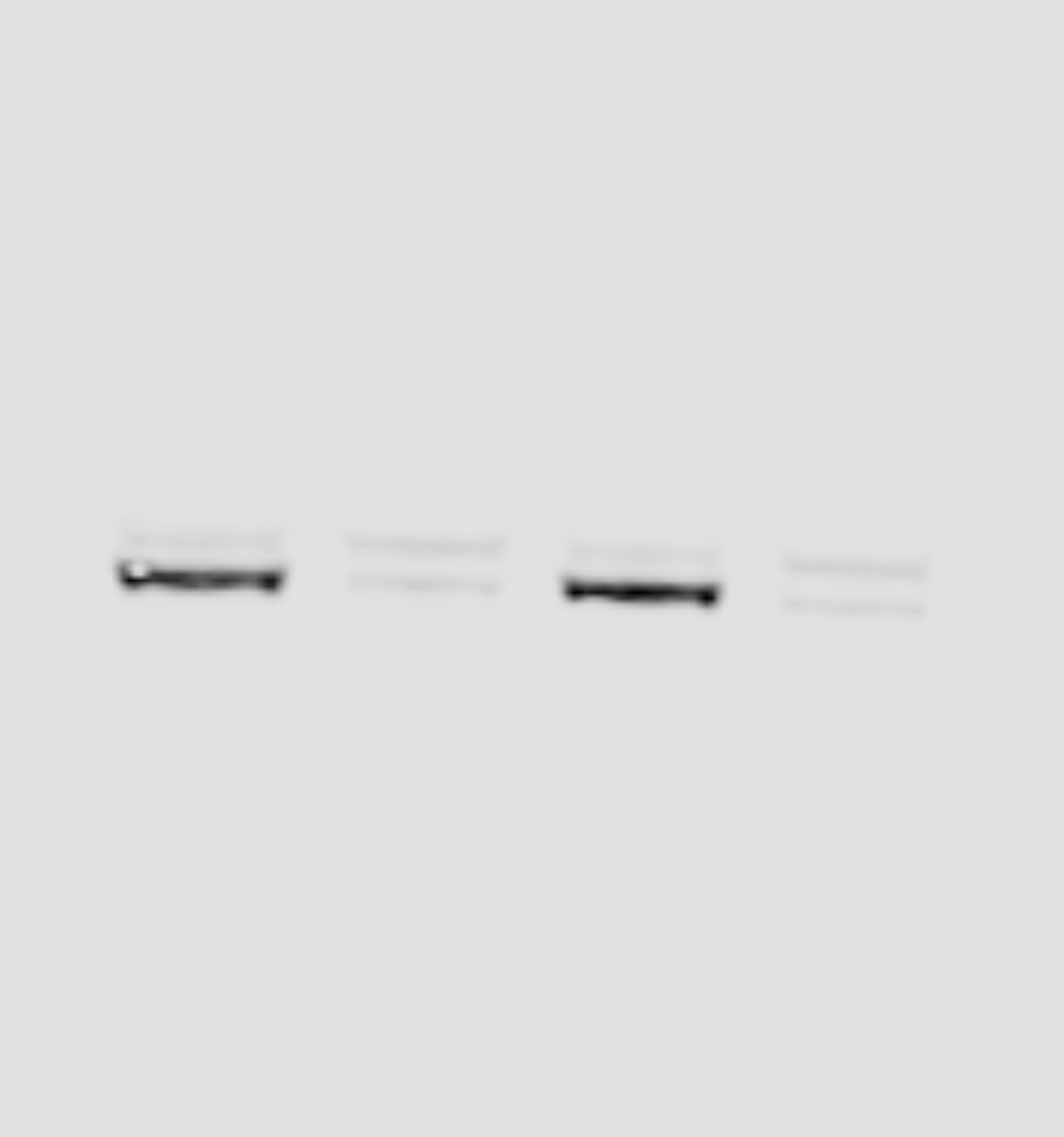

Supplement: Figure 4—figure supplement 1—source data 2. [file elife-107220-fig4-figsupp1-data2.zip › Figure 4-figure supplement 1-source data 2/Figure 4-figure supplement 1-source data 2/Rep 3/Lamin B1.tif]

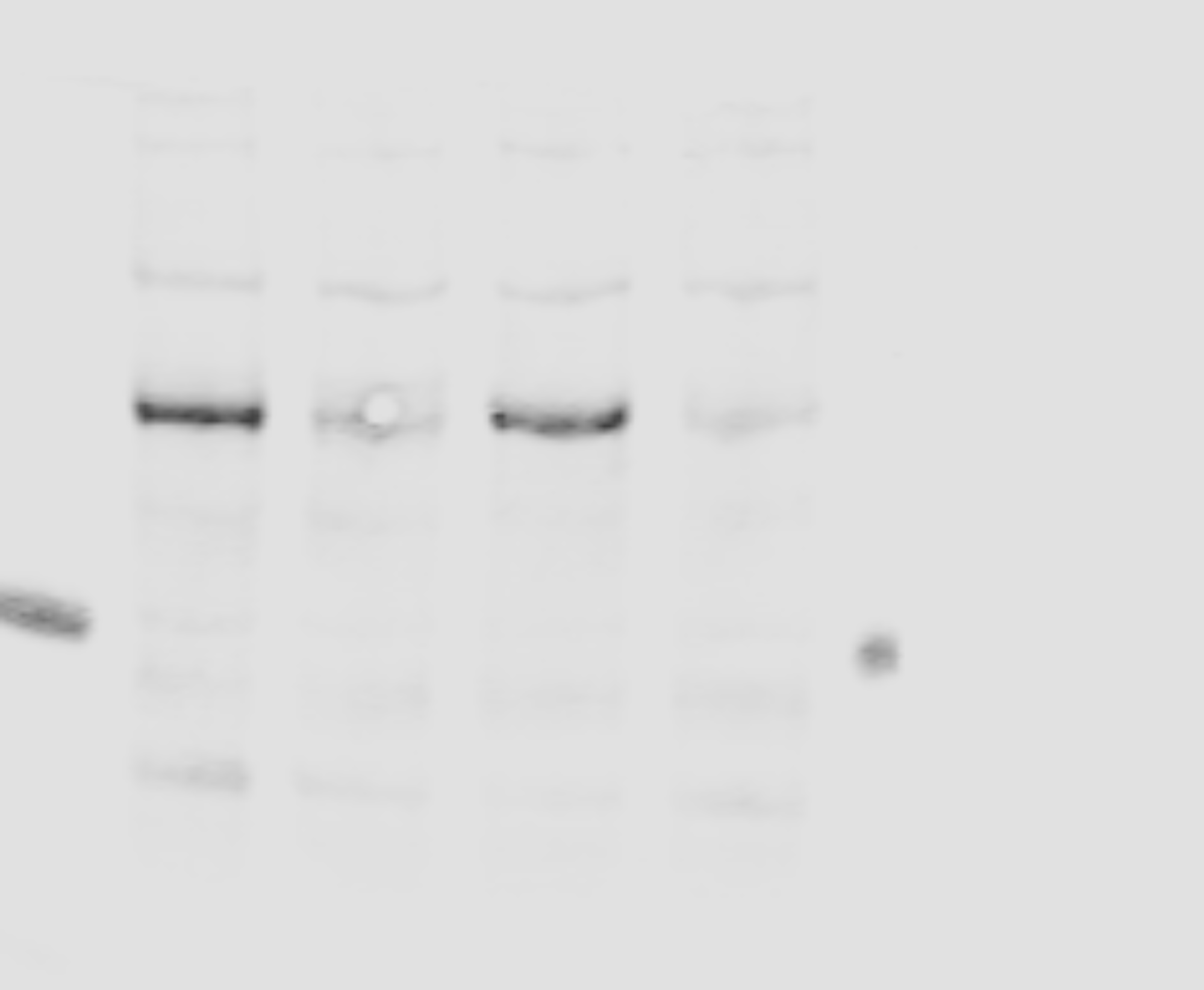

Supplement: Figure 4—figure supplement 1—source data 2. [file elife-107220-fig4-figsupp1-data2.zip › Figure 4-figure supplement 1-source data 2/Figure 4-figure supplement 1-source data 2/Rep 3/Lamin B2.tif]
