# Supplementary material for: Quantifying intracellular mechanosensitive response upon spatially defined mechano-chemical triggering
Source: eLife. 2026 Jun 17;14:RP107220. doi: 10.7554/eLife.107220 (PMC13275063; doi:10.7554/eLife.107220)
Supplement: MDAR checklist [file elife-107220-mdarchecklist1.docx]

**Materials Design Analysis Reporting (MDAR)**

**Checklist for Authors**

The [MDAR framework](https://osf.io/xfpn4/) establishes a minimum set of requirements in transparent reporting mainly applicable to studies in the life sciences.

*eLife* asks authors to **provide detailed information within their article** to facilitate the interpretation and replication of their work. Authors can also upload supporting materials to comply with relevant reporting guidelines for health-related research (see [EQUATOR Network](http://www.equator-network.org/%20)), life science research (see the [BioSharing Information Resource](http://biosharing.org/)), or animal research (see the [ARRIVE Guidelines](http://www.plosbiology.org/article/info:doi/10.1371/journal.pbio.1000412) and the [STRANGE Framework](https://doi.org/10.1038/d41586-020-01751-5); for details, see *eLife*’s [Journal Policies](https://reviewer.elifesciences.org/author-guide/journal-policies)). Where applicable, authors should refer to any relevant reporting standards materials in this form.

For all that apply, please note **where in the article** the information is provided. Please note that we also collect information about data availability and ethics in the submission form.

**Materials:**

| **Newly created materials** | **Indicate where provided: section/figure legend** | **N/A** |
| --- | --- | --- |
| The manuscript includes a dedicated "materials availability statement" providing transparent disclosure about availability of newly created materials including details on how materials can be accessed and describing any restrictions on access. |  | - |
|  |  |  |
| **Antibodies** | **Indicate where provided: section/figure legend** | **N/A** |
| Rabbit anti-lamin A/C (Proteintech, 10298-1-AP, AB_2296961)  Rabbit anti-lamin B1 (Abcam, ab16048, AB_443298)  Rabbit anti-lamin B2 (Abcam, ab155319)  Mouse anti-beta-actin (SantaCruz, sc-47778, AB_626632) | Materials and methods – Immunoblotting; Figure 4-figure supplement 1 |  |
|  |  |  |
| **DNA and RNA sequences** | **Indicate where provided: section/figure legend** | **N/A** |
| Short novel DNA or RNA including primers, probes: Sequences should be included or deposited in a public repository. |  | - |
|  |  |  |
| **Cell materials** | **Indicate where provided: section/figure legend** | **N/A** |
| HeLa FRT/TetR (obtained from T. Maier, University Konstanz, <https://www.nature.com/articles/ncomms5397>)  HeLa Gromeier LMNA KO (https://doi.org/10.7554/eLife.63614) | Materials and methods – Cells Preparation; Generation of lamin knockout (KO) and knockdown (KD) cells |  |
| Primary cultures: Provide species, strain, sex of origin, genetic modification status. |  | - |
|  |  |  |
| **Experimental animals** | **Indicate where provided: section/figure legend** | **N/A** |
| Laboratory animals or Model organisms: Provide species, strain, sex, age, genetic modification status. Provide accession number in repository OR supplier name, catalog number, clone number, OR RRID. |  | - |
| Animal observed in or captured from the field: Provide species, sex, and age where possible. |  | - |
|  |  |  |
| **Plants and microbes** | **Indicate where provided: section/figure legend** | **N/A** |
| Plants: provide species and strain, ecotype and cultivar where relevant, unique accession number if available, and source (including location for collected wild specimens). |  | - |
| Microbes: provide species and strain, unique accession number if available, and source. |  | - |
|  |  |  |
| **Human research participants** | **Indicate where provided: section/figure legend) or state if these demographics were not collected** | **N/A** |
| If collected and within the bounds of privacy constraints report on age, sex, gender and ethnicity for all study participants. |  | **-** |

**Design:**

| **Study protocol** | **Indicate where provided: section/figure legend** | **N/A** |
| --- | --- | --- |
| If the study protocol has been pre-registered, provide DOI. For clinical trials, provide the trial registration number OR cite DOI. |  | - |
|  |  |  |
| **Laboratory protocol** | **Indicate where provided: section/figure legend** | **N/A** |
| No DOI for external step-by-step protocol identified. Detailed procedures described in Materials and methods sections (“FluidFM Probe Preparation”, “Mechanical stimulation”, “Mechanochemical stimulation”, “FLIM Data Analysis”). | Methods throughout |  |
|  |  |  |
| **Experimental study design (statistics details) *** | | |
| **For in vivo studies: State whether and how the following have been done** | **Indicate where provided: section/figure legend. If it could have been done, but was not, write “not done”** | **N/A** |
| Sample sizes (n and N) are reported in figure legends. | Statistical Analysis; Figure legends |  |
| Randomisation |  | **-** |
| Blinding |  | - |
| Inclusion/exclusion criteria |  | - |
|  |  |  |
| **Sample definition and in-laboratory replication** | **Indicate where provided: section/figure legend** | **N/A** |
| Biological replicate numbers are reported in figure legends (typically N = 2–3 independent experiments; n values reported per experiment). Example: “Three biological replicates were utilized for mechano-chemical triggering assays and FLIM-based tension measurements” (Figure 4-figure supplement 1 legend). | Figure legends; Statistical Analysis |  |
| N denotes independent biological replicates; n denotes analyzed cells/samples. | Figure legends; Statistical Analysis |  |
|  |  |  |
| **Ethics** | **Indicate where provided: section/submission form** | **N/A** |
| Studies involving human participants: State details of authority granting ethics approval (IRB or equivalent committee(s), provide reference number for approval. |  | - |
| Studies involving experimental animals: State details of authority granting ethics approval (IRB or equivalent committee(s), provide reference number for approval. |  | - |
| Studies involving specimen and field samples: State if relevant permits obtained, provide details of authority approving study; if none were required, explain why. |  | - |
|  |  |  |
| **Dual Use Research of Concern (DURC)** | **Indicate where provided: section/submission form** | **N/A** |
| If study is subject to dual use research of concern regulations, state the authority granting approval and reference number for the regulatory approval. |  | - |

**Analysis:**

| **Attrition** | **Indicate where provided: section/figure legend** | **N/A** |
| --- | --- | --- |
| Data points were excluded when stable FluidFM probe-cell contact at the specified force could not be maintained due to technical failure or cell escape during the experiment. Exclusion was confirmed using force measurement traces and/or loss of imaging focus during FLIM acquisition. No other exclusion criteria were applied. |  |  |
|  |  |  |
| **Statistics** | **Indicate where provided: section/figure legend** | **N/A** |
| Data normality assessed using Shapiro-Wilk test. Statistical comparisons performed using unpaired two-tailed Student’s t-test; Holm correction applied for multiple comparisons where relevant. Significance threshold set at p < 0.05. Exact p-values reported for selected comparisons. Data presented as mean ± SD or mean ± SEM as specified in figure legends. | Statistical Analysis; Figure legends |  |
|  |  |  |
| **Data availability** | **Indicate where provided: section/submission form** | **N/A** |
| “The datasets generated and analyzed during the current study, including the FLIM image data and the Python scripts developed for the quantification of values, statistical analysis, and data visualization are available in the ETH Zürich Research Collection at <https://doi.org/10.3929/ethz-c-000790250>. Additionally, the force and pressure measurements are available upon request.” | Data Availability |  |
| <https://doi.org/10.3929/ethz-c-000790250> |  |  |
| If reused data is publicly available provide accession number in repository OR DOI, OR URL, OR citation. |  | - |
|  |  |  |
| **Code availability** | **Indicate where provided: section/figure legend** | **N/A** |
| Custom Python scripts used for quantification, statistical analysis, and visualization are available in the ETH Zürich Research Collection at <https://doi.org/10.3929/ethz-c-000790250>. | Data Availability; Statistical Analysis |  |
| <https://doi.org/10.3929/ethz-c-000790250> | Data Availability; Statistical Analysis |  |
| If reused code is publicly available provide accession number in repository OR DOI OR URL, OR citation. |  | - |

**Reporting:**

The MDAR framework recommends adoption of discipline-specific guidelines, established and endorsed through community initiatives.

| **Adherence to community standards** | **Indicate where provided: section/figure legend** | **N/A** |
| --- | --- | --- |
| State if relevant guidelines (e.g., ICMJE, MIBBI, ARRIVE, STRANGE) have been followed, and whether a checklist (e.g., CONSORT, PRISMA, ARRIVE) is provided with the manuscript. |  | -- |

* We provide the following guidance regarding transparent reporting and statistics; we also refer authors to [Ten common statistical mistakes to watch out for when writing or reviewing a manuscript](https://doi.org/10.7554/eLife.48175).

**Sample-size estimation**

- You should state whether an appropriate sample size was computed when the study was being designed
- You should state the statistical method of sample size computation and any required assumptions
- If no explicit power analysis was used, you should describe how you decided what sample (replicate) size (number) to use

**Replicates**

- You should report how often each experiment was performed
- You should include a definition of biological versus technical replication
- The data obtained should be provided and sufficient information should be provided to indicate the number of independent biological and/or technical replicates
- If you encountered any outliers, you should describe how these were handled
- Criteria for exclusion/inclusion of data should be clearly stated
- High-throughput sequence data should be uploaded before submission, with a private link for reviewers provided (these are available from both GEO and ArrayExpress)

**Statistical reporting**

- Statistical analysis methods should be described and justified
- Raw data should be presented in figures whenever informative to do so (typically when N per group is less than 10)
- For each experiment, you should identify the statistical tests used, exact values of N, definitions of center, methods of multiple test correction, and dispersion and precision measures (e.g., mean, median, SD, SEM, confidence intervals; and, for the major substantive results, a measure of effect size (e.g., Pearson's r, Cohen's d)
- Report exact p-values wherever possible alongside the summary statistics and 95% confidence intervals. These should be reported for all key questions and not only when the p-value is less than 0.05.

**Group allocation**

- Indicate how samples were allocated into experimental groups (in the case of clinical studies, please specify allocation to treatment method); if randomization was used, please also state if restricted randomization was applied
- Indicate if masking was used during group allocation, data collection and/or data analysis
