# Supplementary material for: Quantifying intracellular mechanosensitive response upon spatially defined mechano-chemical triggering
Source: eLife. 2026 Jun 17;14:RP107220. doi: 10.7554/eLife.107220 (PMC13275063; doi:10.7554/eLife.107220)
Supplement: Peer review history

# Editor report: eLife Assessment

**DOI:** 10.7554/eLife.107220.3.sa0

**Authors:** Megan C King

**Affiliations:** Yale School of Medicine United States

**Keywords:** Convincing, Valuable

This **valuable** study provides evidence that the integration of the nuclear envelope into the endoplasmic reticulum provides a mechanism for mechanical integration across this continuous membrane system. This work opens up new avenues for studying organelle membrane tension homeostasis. The evidence was found to be **convincing** and carefully quantified, with minor limitations that we expect to be further explored in future work.

# Referee report: Reviewer #1 (Public review)

**DOI:** 10.7554/eLife.107220.3.sa1

Summary:

Zare‑Eelanjegh et al. investigate how the endoplasmic reticulum, the nucleus, and the cell periphery are mechanically linked by indenting intact cells with specially shaped atomic‑force probes that double as drug injection devices. Fluorescence‑lifetime imaging of the membrane tension reporter Flipper‑TR reveals that these three compartments are mechanically linked and that the actin cytoskeleton, microtubules, and lamins modulate this coupling in complex ways.

Strengths:

* The study makes an important advance by applying FluidFM to probe organelle mechanics in living cells, a technically demanding but powerful approach.

* Experimental design is quantitative, the data are clearly presented, and the conclusions are broadly consistent with the measurements.

Weaknesses:

* Calcium‑dependent effects: Indentation can evoke cytoplasmic Ca²⁺ elevations that drive myosin contraction and reshape the internal membrane network (e.g., vesiculation: PMID : 9200614, 32179693) possibly confounding the Flipper-TR responses; without simultaneous/matching Ca²⁺ imaging, cell viability assays (e.g., Sytox), and intracellular Ca²⁺ sequestration or myosin inhibition experiments, a more complex mechanochemical coupling cannot be excluded, weakening conclusions.

* Baseline measurements: Flipper‑TR lifetime images acquired without indentation do not exclude potential light‑induced or time‑dependent changes, which weakens the conclusions.

* Indentation depth versus nuclear stiffness/tension: Because lamin‑A/C depletion softens nuclei, a given force may produce a deeper pit and thus greater membrane stretch. It is unclear how the cytoskeletal perturbations affect indentation depth, which weakens the conclusions.

Comments on revisions:

With their responses, the authors have relieved my initial concerns.

# Referee report: Reviewer #2 (Public review)

**DOI:** 10.7554/eLife.107220.3.sa2

Summary

This valuable study combines atomic force microscopy with genetic manipulations of the lamin meshwork and microinjection of cytoskeletal depolymerizing drugs to probe the mechanical responses of intracellular organelles to combinations of cytoskeletal perturbations. This study demonstrates both local and distal responses of intracellular organelles to mechanical forces, and shows that these responses are affected by disruption of the actin, microtubule, and lamin cytoskeletal systems.

Strengths:

This study uses a sensitive micromanipulation system to apply and visualize the effects of force on intracellular organelles.

# Author comment: Author response

**DOI:** 10.7554/eLife.107220.3.sa3

**Authors:** Elaheh Zare-Eelanjegh, Renard TM Lewis, Ines Lüchtefeld, Ulrike Kutay, Tomaso Zambelli

**Affiliations:** ETH Zurich Zurich Switzerland; ETH Zurich Zurich Switzerland; ETH Zurich Zurich Switzerland; ETH Zurich Zurich Switzerland; ETH Zurich Zurich Switzerland

The following is the authors’ response to the original reviews.

**Public Reviews:**

**Reviewer #1 (Public review):**

Summary:

Zare-Eelanjegh et al. investigate how the endoplasmic reticulum, the nucleus, and the cell periphery are mechanically linked by indenting intact cells with specially shaped atomic force probes that double as drug injection devices. -Fluorescencelifetime imaging of the membrane tension reporter -FlipperTR- reveals that these three compartments are mechanically linked and that the actin cytoskeleton, microtubules, and lamins modulate this coupling in complex ways.

Strengths:

(1) The study makes an important advance by applying FluidFM to probe organelle mechanics in living cells, a technically demanding but powerful approach.

(2) Experimental design is quantitative, the data are clearly presented, and the conclusions are broadly consistent with the measurements.

Weaknesses:

(1) Calcium-dependent- effects: Indentation can evoke cytoplasmic CA^2+^ elevations that drive myosin contraction and reshape the internal membrane network (e.g., vesiculation: PMID : 9200614, 32179693) possibly confounding the Flipper-TR responses; without simultaneous/matching CA^2+^ imaging, cell viability assays (e.g., Sytox), and intracellular CA^2+^ sequestration or myosin inhibition experiments, a more complex mechanochemical coupling cannot be excluded, weakening conclusions.

(2) Baseline measurements: FlipperTR lifetime images acquired without indentation do not exclude potential -light-induced or -time-dependent- changes, which weaken the conclusions.

(3) Indentation depth versus nuclear stiffness/tension: Because lamin-A/C depletion softens nuclei, a given force may produce a deeper pit and thus greater membrane stretch. It is unclear how the cytoskeletal perturbations affect indentation depth, which weakens the conclusions.

**Reviewer #2 (Public review):**

Summary:

This useful study combines atomic force microscopy with genetic manipulations of the lamin meshwork and microinjection of cytoskeletal depolymerizing drugs to probe the mechanical responses of intracellular organelles to combinations of cytoskeletal perturbations. This study demonstrates both local and distal responses of intracellular organelles to mechanical forces and shows that these responses are affected by disruption of the actin, microtubule, and lamin cytoskeletal systems. Interpretation of these effects is limited by the absence of key data determining whether acute microinjection of cytoskeleton-depolymerizing drugs has complete or partial effects on the targeted cytoskeletal networks.

Strengths:

This study uses a sensitive micromanipulation system to apply and visualize the effects of force on intracellular organelles.

Weaknesses:

The choice to deliver cytoskeleton-depolymerizing drugs by local microinjection is unusual, and it is unclear to what extent actin and microtubule filaments are actually depolymerized immediately after microinjection and on the minutes-length timescale being evaluated in this study. This omission limits the interpretation of these data.

**Reviewer #3 (Public review):**

Summary:

Using an approach developed by the authors (FluidFM) combined with FLIM, they discover that a mechanical force applied over the cell nucleus triggers mechanical responses dependent on the Lamina composition.

Strengths:

The authors present a new approach to study mechano-transduction in living cells, with which they uncover lamin-dependent properties of the nucleus.

Weaknesses:

(1) The transfer of the mechanical response from the Lamina to the ER is not fully covered.

(2) In Figure 4D, WT dots are the same for each compartment. Why do the authors not make one graph for each compartment with WT, A-KO, B-KD, and A-KO/B-KD together?

(3) In Figure 1E, the authors showed well how the probe deforms the nucleus. It is not indicated in the material and methods section or in the figure legend, where, in Z, the acquisition of FLIM images was made or if it is a maximum projection. I assume it was made at a plane in the middle of the nucleus to see the nuclear envelope border and the ER at the same time. Did the authors look at the nuclear membrane facing upward, where most of the deformation should occur? Are there more lifetime changes? In Figure D, before injection of CytoD, we can clearly see a difference at the pyramidal indentation site with two different lifetime colors.

(4) A great result of this article regards the importance of Lamins, A and B, in triggering the response to a mechanical force applied to the nucleus. Could 3D imaging for LaminA and LaminB be performed at the different time points of indentation to see how the lamins meshworks are deformed and how they return to basal state? This could be correlated with the FLIM results described in the article.

(5) Lamins form a meshwork underneath the nuclear membrane. They are connected to the cytoskeletons mainly by the LINC complex. Results presented here show that the cytoskeletons are implicated in transferring the stimulus from the nuclear envelope to the ER. Could the author perform the same experiments using Nesprin-2 or/and Nesprin-1 or/and SUN1/2 knockdowns to determine if this transmission is occurring through the LINC complex or rather in a passive way by modifying the nuclear close surroundings?

(6) The authors used cytoskeleton drugs, CytoD and Nocodazole, with their FluidFM probe, but did not show if the drugs actually worked and to what extent by performing actin or microtubule stainings. In the original paper describing FluidFM, 15s were enough to obtain a full FITC-positive cell after injection. Here, the experiments are around 5 minutes long. I therefore interrogate the rationale behind the injection of the drugs compared to direct incubation, besides affecting only the cell currently under indentation.

We thank the reviewers for their constructive criticisms and suggestions. Accordingly, we amended the manuscript and the figures.

**Recommendations for the authors:**

**Reviewer #1 (Recommendations for the authors):**

(1) Calcium-dependent effects: Indentation can evoke cytoplasmic CA^2+^ elevations that drive myosin contraction and reshape the internal membrane network (e.g., vesiculation: PMID : 9200614, 32179693) that may affect Flipper-TR signals independent of membrane tension; without simultaneous CA^2+^ imaging, cell viability assays (e.g., Sytox costaining), intracellular CA^2+^ sequestration or myosin inhibition, a more complex mechanochemical coupling cannot be excluded. Tracking ER morphology during the experiments with luminal and membrane markers would further clarify this point.

For the goal of our article which is exhibiting and quantifying the tension propagation and tension homeostasis over different organelles managing the mechanosensitivity and thus the mechanoresponse of cell, the test cells (drug injected cells) were compared with the control group of non-drug injected cells (Fig. 2 and Fig. 3), and in these cases potential overall responses of the cells to intendation, e.g. potential changes in CA^2+^ sequestration, are covered by the control group.

Interestingly, using only cylindrical probes in CytoD injection while indenting cells, demonstrated higher tension at the NE compared to the control group of non-drug injected cells. This indicates that a higher effect arising from the F-actin-disturbance phenomena compared to the indention process itself, at least where the cells were stimulated using cylindrical probes. That was also the reason why in the next steps of this study including varying the indentation site from the nucleus to the ER or cell periphery as well as studying WT cells compared to varied lamina compositions, only cylindrical probes with minimized indention effect on the NE and the ER were used.

Lastly, to examine simultaneously response to tension changes and calcium dynamics, we have meanwhile extended our study and analyzed cells treated with different cytoskeleton disturbing drugs (e.g., CytoD), subjected to viscoelasticity measurements using AFM indentation (i.e. cells relaxation studies following indentation), and injected with drugs perturbing the regulation of CA^2+^ homeostasis (i.e., Thapsigargin), combined with simultaneous CA^2+^ imaging, for which another manuscript is in preparation.

(2) Baseline measurements: FlipperTR lifetime images acquired without indentation, collected with identical timing and illumination, are needed as controls to gauge potential light-induced or time-dependent changes.

For every cell a baseline referring to its tension at relaxed state (without indentation) was quantified by a Flipper-TR image taken before the indention and injection processes (“before”). As explained in the manuscript (lines 180-184), this baseline tension value was then used to be subtracted from the tension measured over time by the time-lapse FlipperTR imaging over the course of 3-4 min of stimulation (indentation + injection) as well as immediately or 5 min post-stimulus. The control group (i.e., non-drug injected cells or WT cells where the effect of F-actin depolymerization or the effect of lamina composition were studied, respectively) was always performed in the same manner as for test group. As such, tenson increase due to the light-inducing, time-dependent changes or indentation solely, were excluded.

(3) Indentation depth versus nuclear stiffness/tension: Because laminA/C depletion softens nuclei, a given force may arguably produce a deeper pit and thus greater (not less) membrane stretch. Demonstrating that pit geometry depends only on applied force - and not on genetic or pharmacological perturbations - is necessary to rule out alternative interpretations.

We thank the reviewer for raising this important point regarding the relationship between indentation depth and nuclear stiffness. To address whether pit geometry depends on applied force rather than genetic perturbations, we analyzed the piezo movement required to reach the 150 nN force setpoint across all experimental conditions (WT, *LMNA* KO, LMNB KD, and *LMNA* KO/LMNB KD cells).

Our results (Fig. S6) demonstrate that there is no statistically significant difference in the piezo displacement from the contact point to the 150 nN setpoint between any of the experimental groups (Kruskal-Wallis H-test: H = 1.744, p = 0.627). This indicates that for a constant applied force of 150 nN, the indentation depth is equivalent across all conditions despite differences in nuclear stiffness.

Therefore, the observed differences in tension response and perhaps the membrane stretch cannot be attributed to variations in indentation depth but rather reflect the intrinsic differences in molecular mechanical response to equivalent mechanical stimuli.

This has been added in the manuscript in lines 282-286.

**Reviewer #2 (Recommendations for the authors):**

(1) Please clarify the distinctions between the pyramidal and cylindrical probes. The manuscript alludes to sharpening the cylindrical probe to facilitate membrane rupture. Do both probes rupture the plasma membrane upon force application? If so, at what applied force does this occur? It seems that PM rupture would also affect tension on intracellular membranes during and especially after force application.

Yes, both cylindrical and pyramidal probes are rupturing PM as well as the nuclear membrane when targeting the nucleus of cells. When targeting Hela cells, used for this study, pyramidal probes puncture the membrane at a higher force of 100 nN compared to rupture forces between 10 nN and 50 nN required for sharpened cylindrical probes used here. This was explained in manuscript lines 112-115 for cylindrical probes and revised for pyramidal probes in lines 115-119.

(2) Also re: probes: it is clear from Figure 1 that the total volume displacement induced by the pyramidal probe is far greater than the cylindrical probe. This greater displaced volume seems to be a very reasonable explanation for the increased membrane tension detected with the pyramidal probe, but this interpretation is not discussed.

That is a good point, thank you! This has been added in lines 138-140.

(3) Both cytochalasin D and nocodazole work by preventing new polymerization of monomers, which acutely affects new assembly and, over time, leads to loss of polymerized filaments. On the timescale of the experiments shown, it seems possible that acute effects on new filament assembly may be occurring, but that pre-assembled filaments may remain stable. It may thus be a misinterpretation to describe these conditions as "without actin fibers" or "without MTs". Further complicating matters, it is possible that the kinetics of filament disassembly may be altered by combinatorial treatment and/or in lamin knockout conditions versus wild-type cells. For instance, it has been shown that microtubule depolymerization increases actin contractility (see PMID 33089509). For these reasons, control experiments showing the extent of actin and/or microtubule disassembly in each condition tested are essential to interpret the data shown.

Thank you for rasing this valid point. This has been corrected and noted as "less actin fibers" and "less MTs". For what concerns the timescale within which the drugs (*e.g.*, CytoD and Nocodazole) affect the filaments assembly, a higher concentration of 50 µM for each of CytoD and Nocodazole leading to final concentration of 0.5 µM was used for intracellular injection. This final physiologically relevant concentration was expected to act as fast as 12 min for CytoD and 1-5 min for Nocodazole when directly delivered inside the cell, excluding the required time for passing the plasma membrane. Especially in our study examining the dynamic response of cells and change in tension is focusing on the early effects of drugs and deviation from the control groups rather than the steady state achieved at longer time points. The basis for the time estimation relies on the reported values in the literature. For instance, a recent comprehensive study quantified actin dynamics and its interaction with CytoD using high resolution images of single actin filaments acquired by total internal reflection fluorescence (TIRF) microscopy and reported a value of approximately 150 s (depicted from the graphs presented in Fig. 2D and 2F) as a starting point of inhibiting actin filaments polymerization after introducing 5 nM CytoD flow in a chamber containing actin filaments.1 Or in another study, a half-time of 40 s for the complete disassembly of microtubules in monocytes has been reported for cells incubated with 1 µM Nocodazole.2 This part was also included in SI file, section “Mechanochemical stimulation”.

(4) The presentation of some of the data could be clarified. For instance, it is unclear how some time course experiments can be non-significant but the endpoint analysis can be significant (for instance, Figure 3C vs. Figure 3D.)

We agree that some instances require clearer interpretation: indenting cell nucleus using cylindrical probes induced a higher tension at CytoD-injected cells compared to control cells at both the ER and NE, during and after stimulus (Fig. 2E-F and Fig. 3C-D). Time lapse tension analysis of these cells at the ER and NE showed a close to significant and significant differences between test and control groups, respectively. *p*-values of 0.087 for Fig. 2E (bottom row, ER) and 0.042 for Fig. 3C (top row, ER) were captured at the ER for the last time point during stimulus. For “after stimulus” condition, significant differences between CytoD-injected and control cells at both the ER and NE were captured. The ER’s complex morphology consists of many curved structures of lumens and disks which can deform when subjected to external mechanical perturbation, making it prone to absorb stress and strain when directly targeted. That could explain the similar tension levels in both CytoD-injected and control cells during ER indentation. Notably, unlike nucleus-targeted cells, ER-targeted cells only show increased tension at the ER and NE in CytoDinjected cells compared to control ones after stimulation. This suggests fundamental differences in the mechanical coupling of the nucleus and the ER to the cytoskeleton. While the nucleus maintains direct, structural actin connections through the nuclear lamina and LINC complexes3, making it immediately sensitive to actin disruption, the ER relies on indirect, signaling-mediated cytoskeletal interactions4,5. Thus, the ER functions as a dynamic tension buffer that engages cytoskeletal support primarily during active repair processes following mechanical perturbation. This explains why nuclear probing reveals immediate tension differences in actin-disrupted cells, while ER probing only shows post-retraction effects. Consequently, statistical analysis detects significant differences between test and control groups after probe removal, but not during probe contact in ER-targeted experiments. This was also explained better in the manuscript in line 236.

References

(1) Mitani, T. et al. Microscopic and structural observations of actin filament capping and severing by Cytochalasin D. bioRxiv, 2025.2001.2028.635382 (2025).

(2) Cassimeris, L. U., Wadsworth, P. & Salmon, E. D. Dynamics of microtubule depolymerization in monocytes. J Cell Biol 102, 2023-2032 (1986).

(3) Maurer, M. & Lammerding, J. The Driving Force: Nuclear Mechanotransduction in Cellular Function, Fate, and Disease. Annu Rev Biomed Eng 21, 443-468 (2019).

(4) Shi, X. et al. Actin nucleator formins regulate the tension-buffering function of caveolin-1. J Mol Cell Biol 13, 876-888 (2022).

(5) van Vliet, A. R. & Agostinis, P. PERK and filamin A in actin cytoskeleton remodeling at ER-plasma membrane contact sites. Molecular & Cellular Oncology 4, e1340105 (2017).
